# A hypothetical astrocyte–microglia lactate shuttle derived from a ^1^H NMR metabolomics analysis of cerebrospinal fluid from a cohort of South African children with tuberculous meningitis

**DOI:** 10.1007/s11306-014-0741-z

**Published:** 2014-10-11

**Authors:** Shayne Mason, A. Marceline van Furth, Lodewyk J. Mienie, Udo F. H. Engelke, Ron A. Wevers, Regan Solomons, Carolus J. Reinecke

**Affiliations:** 1 Centre for Human Metabonomics, Faculty of Natural Sciences, North-West University (Potchefstroom Campus), Private Bag X6001, Potchefstroom, 2531 South Africa; 2 Department of Paediatric Infectious Diseases–Immunology and Rheumatology, Vrije Universiteit Medical Centre, De Boelelaan 1117, 1081 HV Amsterdam, The Netherlands; 3 Potchefstroom Laboratory for Inborn Errors of Metabolism, Division for Biochemistry, North-West University (Potchefstroom Campus), Private Bag X6001, Potchefstroom, South Africa; 4 Department of Laboratory Medicine, Radboud University Nijmegen Medical Centre, PO Box 9101, 6500 HB Nijmegen, The Netherlands; 5 Department of Paediatrics and Child Health, Faculty of Medicine and Health Sciences, Stellenbosch University, PO Box 19063, Tygerberg, 7505 South Africa

**Keywords:** Tuberculous meningitis (TBM), Cerebrospinal fluid (CSF), Nuclear magnetic resonance (NMR) metabolomics, “Astrocyte–microglia lactate shuttle” (AMLS) hypothesis

## Abstract

Tuberculosis meningitis (TBM) is the most severe form of extra-pulmonary tuberculosis and is particularly intense in small children; there is no universally accepted algorithm for the diagnosis and substantiation of TB infection, which can lead to delayed intervention, a high risk factor for morbidity and mortality. In this study a proton magnetic resonance (^1^H NMR)-based metabolomics analysis and several chemometric methods were applied to data generated from lumber cerebrospinal fluid (CSF) samples from three experimental groups: (1) South African infants and children with confirmed TBM, (2) non-meningitis South African infants and children as controls, and (3) neurological controls from the Netherlands. A total of 16 NMR-derived CSF metabolites were identified, which clearly differentiated between the controls and TBM cases under investigation. The defining metabolites were the combination of perturbed glucose and highly elevated lactate, common to some other neurological disorders. The remaining 14 metabolites of the host’s response to TBM were likewise mainly energy-associated indicators. We subsequently generated a hypothesis expressed as an “astrocyte–microglia lactate shuttle” (AMLS) based on the host’s response, which emerged from the NMR-metabolomics information. Activation of microglia, as implied by the AMLS hypothesis, does not, however, present a uniform process and involves intricate interactions and feedback loops between the microglia, astrocytes and neurons that hamper attempts to construct basic and linear cascades of cause and effect; TBM involves a complex integration of the responses from the various cell types present within the CNS, with microglia and the astrocytes as main players.

**Electronic supplementary material:**

The online version of this article (doi:10.1007/s11306-014-0741-z) contains supplementary material, which is available to authorized users.

## Introduction

Tuberculosis (TB), one of the major contemporary pandemics, is caused by *Mycobacterium tuberculosis* (Mtb); its worldwide impact remains a serious concern. The disease in children is particularly severe as diagnosis is difficult since its identification in children usually results from a combination of, often asymptomatic, clinical criteria, non-specific TB tests and diagnostic markers, all of which have a low sensitivity and specificity (van Well et al. 2009), leading to delayed intervention, and a high risk of morbidity and mortality. According to the WHO Global TB Report for 2012 (WHO 2013), there were approximately 325,000 clinically defined new cases of TB in South Africa alone, of which 42,000 (14 %) were of the extra-pulmonary forms (EPTB). Central nervous system (CNS) TB accounts for an estimated 1–10 % of all EPTB (Cherian and Thomas 2011; Bhigjee et al. 2007; Rock et al. 2005), with tuberculous meningitis—also known as TB meningitis (TBM)—being not only the most severe complication of the disease but also the most common form of bacterial meningitis (BM) in South African children (Wolzak et al. 2012). Infants and young children are particularly prone to dissemination of TB, especially TBM, due to their young age and immature immune systems. Most TBM patients upon admission present with an advanced stage of disease due to difficulty of diagnosis in the early stages, resulting in an estimated 30 % of patients dying despite treatment (Youssef et al. 2006).

Cerebrospinal fluid (CSF) is the biofluid that is in direct contact with the site of TBM infection and contains a wealth of information potentially useful for TBM diagnosis and treatment. Evaluation of the characteristics of CSF is already a key factor in the diagnosis of several CNS diseases (Seehusen et al. 2003; Watson and Scott 1995), particularly of TBM (van Well et al. 2009). CSF from TBM cases typically shows a clear appearance, moderate pleocytosis with a predominance of lymphocytes, increased protein content and a very low glucose concentration (Principi and Esposito 2012). To improve on these general clinical observations, more sophisticated clinical-chemistry tests on CSF have been proposed but none with elevated sensitivity and specificity (Ho et al. 2013), although potentially useful to support the diagnosis in some cases (Principi and Esposito 2012). Improved clinical outcomes are highly dependent on timely diagnosis and initiation of appropriate treatment for survival benefit (Ruslami et al. 2013), leaving monitoring of treatment likewise a key developmental need for this complex infectious disease, to which an untargeted metabolomics investigation could make a distinct contribution, as shown in this paper.

Metabolomics information has the advantage of being context dependent by disclosing the complement of metabolites associated with a physiological, developmental, or pathological state prevailing in a cell, tissue, organ, or organism. Infection of the Mtb bacilli occurs through inhalation of aerosolized droplets and harbouring of the bacilli in the pulmonary system. Should dissemination into the lympho-hematogenous circulatory system occur (Krishnan et al. 2010; El-Kebir et al. 2013), the brain becomes a very attractive site for the establishment of metastatic foci due its rich vascular and oxygen supply. Mtb appears to enter the subarachnoid space via rupture of an adjacent parenchymal tubercle (Leonard and Des Prez 1990) and CD14 receptors of the microglia may render them as the principal cell target in the CNS (Peterson et al. 1995). Microglial cells are the resident macrophages of the brain parenchyma; they share many, if not all, of the properties of macrophages in other tissues (Nareika et al. 2005), such as production of a variety of cytokines and chemokines on activation. Despite this hostile bactericidal environment, it provides protection and shelter to Mtb from other immune cells and promotes development of small tuberculous foci, commonly known as granulomas (Ernst 1998; Davis and Ramakrishnan 2009), presenting one of the ultimate paradoxes in the study of TBM host–pathogen interactions (Thi et al. 2012).

A unique aspect of TB in children is the unnoticed infection by Mtb in the often rapid development of the disease (Swaminathan and Rekha 2010), usually in children younger than 5 years and/or in HIV-infected children (Starke 2003). The outcome of TBM correlates with the stage at which disease treatment starts, underlining the need for early detection and a prompt clinical response. Metabolomics proved to be suited for a powerful global study of diseases of the human CNS, with the potential of mapping early biochemical changes in these diseases, providing opportunities for timely diagnosis and development of signatures and hence for assisting in effective interventions (Dunne et al. 2005; Kaddurah-Daouk and Krishnan 2009; Madsen et al. 2010; Sinclair et al. 2009). The systematic characterization of the CSF metabolome is well established with contributions coming from studies using various platforms (Guo et al. 2011; Mandal et al. 2012; Stoop et al. 2010; Wevers et al. 1995; Wishart et al. 2008). To date, the presence of 476 detectable metabolites with an extensive dynamic range has been confirmed in the human CSF metabolome, of which 170 are readily measurable, 75 have concentrations above 1 µM and 47 metabolites have been quantified by NMR by Wishart et al. (2008). Due to its hydrophilic nature, NMR can be considered the best platform for characterizing CSF. Major challenges for investigations of the CSF metabolome are (1) the diverse nature and low concentrations of metabolites, (2) limited availability and (3) small sample volumes (Guo et al. 2011; Stoop et al. 2010), as a result of ethical issues, such as pain and health risks, that restrict the general availability of CSF for research purposes (Geiszler et al. 2013). To date, NMR-based metabolomics of TB has been conducted on infected mice (Shin et al. 2011), guinea pigs (Somashekar et al. 2011) and humans (Zhou et al. 2013), on other forms of meningitis (Coen et al. 2005; Himmelreich et al. 2009) and a study by Subramanian et al. (2005), which selected 12 CSF metabolites for differential diagnosis of meningitis in children through in-house software diagnostics.

The focus of the NMR metabolomics study of CSF reported here is directed towards the host’s metabolic response to the TBM infection. The unusually high incidence of TB in childhood in isolated regions in the Western Cape province of South Africa provided a unique opportunity to obtain CSF samples for a metabolomics study based on a cohort of infants and small children definitively diagnosed with TBM. Two control groups were likewise used in this investigation: the first consisted of South African infants and children initially suspected to be suffering from meningitis (but subsequently shown to be negative), and secondly, control samples from an unrelated neurological study in the Netherlands. The outcome of this NMR investigation revealed metabolic perturbations due to TBM, and has considerable potential for improving the early clinical assessment of the disease state in TBM and for monitoring responses to treatment regimes. The metabolic profile also contributed to hypothesis formulation, proposed as an “astrocyte–microglia lactate shuttle” (AMLS), encapsulating the unique metabolic plasticity in the CNS metabolism towards the energy requirements for the microglia-driven neuroinflammatory responses, of which TBM may be one such example.

## Materials and methods

### Experimental design

**Fig. 1:**
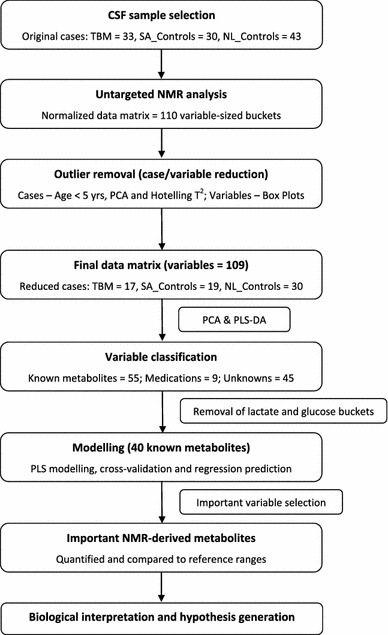
Schematic representation of the work flow following data generation, based on statistical pre-processing, data analysis and cross-validation to identify important NMR-derived metabolites of TBM infection

Metabolic fingerprinting of NMR data requires pre-processing to generate a data matrix of variables and cases of an operational size, to be followed by multivariate analysis to identify the relevant analytical information. The work flow that we followed is shown schematically in Fig. 1. Our selected experimental group consisted of 33 children with confirmed TBM and two control groups, as described below. Untargeted ^1^H NMR analysis of CSF samples yielded spectra that were resolved into 110 buckets of variable size. Following removal of outliers from the patient and two control groups, the important, known metabolites observed in the TBM-infected cases were identified and cross-validated by statistical modelling. The 16 NMR-derived metabolites differentiating TBM from non-TBM, based on this South African cohort of patients, were then quantified, compared to normal reference ranges and the corresponding biological interpretation given, from which our hypothesis was generated.

### Sample collection, description and storage

The three experimental groups used in this study were: (1) South African patients with confirmed TBM (see comprehensive description of clinical profiles in section S1, Table S1 and Table S2 of the Supplementary Information (SI), and on the medication used for treatment in Table S3); (2) non-meningitis South African controls (SA_Controls); and (3) neurological controls from the Netherlands (NL_Controls). The first two groups comprised children between the ages of 6 months and 12 years, all of whom were originally suspected meningitis cases and referred from local clinics to the paediatrics unit at Tygerberg Hospital in the Western Cape province of South Africa. In most of these cases, a broad range of non-specific treatments (e.g., broad-spectrum antibiotics, analgesics and anti-inflammatories) were initiated prior to admission. These individuals were given a thorough assessment by a paediatric neurologist at Tygerberg Hospital, which involved an extensive description of the clinical background, including polymerase chain reaction and culture analysis of a CSF sample obtained through a lumbar puncture. The main physical presentations and clinical symptoms of these cases, as testified by parents and observed by the respective clinicians, were compatible with non-CNS indications of TB outside the CNS, typically observed in small TB-infected children from the region. As part of the diagnostic process a detailed inspection of the clinical chemistry of the CSF was conducted and yielded a report describing the count and type of cells present, particularly immune response-related cells (Table S2), as well as a measure of CSF protein (mostly high: >1 g/L) and glucose (mostly low: <2.2 mmol/L or CSF:blood glucose ratio <50 %) levels. The South African controls were confirmed negative for any form of meningitis, despite being ill with symptoms reminiscent of the condition. Ethical and practical considerations limited the availability of healthy controls, which could partially be overcome through a comparative analysis of a second control group with CSF collected from untreated individuals from the Radboud University Medical Centre in Nijmegen, the Netherlands. The Netherlands non-TBM control group (the third experimental group) consists of CSF samples from age-matched patients who were suspected to suffer from a neurometabolic disease. After appropriate and in depth investigations no clinical or biochemical evidence was found for such diagnosis in any of these patients. The only disease exclusion criterion applied to all cases was HIV co-infection.

The present study was approved by the Human Research Ethics Committee of Stellenbosch University, South Africa (study nr. N11/01/006).

### Sample preparation and ^1^H NMR spectroscopy

All CSF samples were stored at −80 °C prior to analysis and transported to the NMR facility at the Laboratory for Genetic, Endocrine and Metabolic Diseases at the Radboud University Medical Centre in Nijmegen, where the NMR analyses were conducted. Sample preparation followed the standard operating procedure as established by the Nijmegen laboratory (Engelke et al. 2005, 2006; Wevers et al. 1995).

Each CSF sample was measured at 500 MHz on a Bruker DRX Avance spectrometer equipped with a triple-resonance inverse probe head. Software used was: Bruker Topspin (V3.1) for data pre-processing and Bruker AMIX (V3.9.12) for binning and quantification (Ellinger et al. 2013). Variable-sized bucketing was used to create a data matrix of 110 variables, followed by log transformation. The NMR region representing the suppressed water signal (4.67–4.96 ppm) was excluded from the data matrix. A more detailed description of the NMR protocol and pre-treatment of the original NMR-data are included in section S2 and S3, respectively, of the SI.

### Statistical analyses

The original TBM group was slightly reduced by excluding 4 cases which were older than 5 years of age, intended to define a more homogeneous group of infants and children. Unsupervised principal component analysis (PCA) and Hotelling’s T^2^ test, with a confidence level of 95 %, was used to remove further case outliers for each experimental group; assessment of overall variance of variables was used to remove variable outliers. The multivariate analyses (see Fig. 1) were done using the statistical software package The Unscrambler^®^ X (V10.3, CAMO software AS, Norway), successfully applied in other metabolomics studies (e.g., Maddula and Baumbach 2011). Supervised partial least squares-discriminant analysis (PLS-DA) modelling was used to identify the important variables and cross-validated by leaving one sample out at a time, using the same samples for model estimation and testing. The principal advantage of this leave-one-out cross-validation is that it is an efficient method of dealing with a small number of cases (as is typically the situation with metabolomics data) and allows for the jack-knifing approach on which an uncertainty test is based. The uncertainty test creates a number of sub-models on all the samples not kept in the cross-validation segment; for every sub-model the variations are estimated to assess the stability of the results and an overall *p* value is output per validated variable based on the PLS model. Following this, a regression model is given that plots the predicted Y-values of the PLS model for all samples, together with a deviation that expresses the uncertainty of the prediction. The deviations are estimated as a function of the global model error, the sample leverage, and each sample’s residual X-variance. A small deviation indicates that the sample used for prediction is similar to the samples used for the calibration model, whereas predicted Y-values with high deviations are less reliable. This predicted regression model is used to assess how well the PLS model is able to correctly classify cases as TBM or non-TBM.

The online metabolomics suite, Metaboanalyst 2.0 (www.metaboanalyst.ca) (Xia et al. 2009), was used to create the quantitative VIP values [Variables Important in Projection (VIP) are conventionally used in metabolomics studies to identify important metabolites] for the PLS-DA analyses, as well as to calculate the univariate measures [fold change (d-values) and *t* test (*p* values)] for the final reduced data matrix.

## Results and discussion

### Data analysis and identification of important variables

**Fig. 2:**
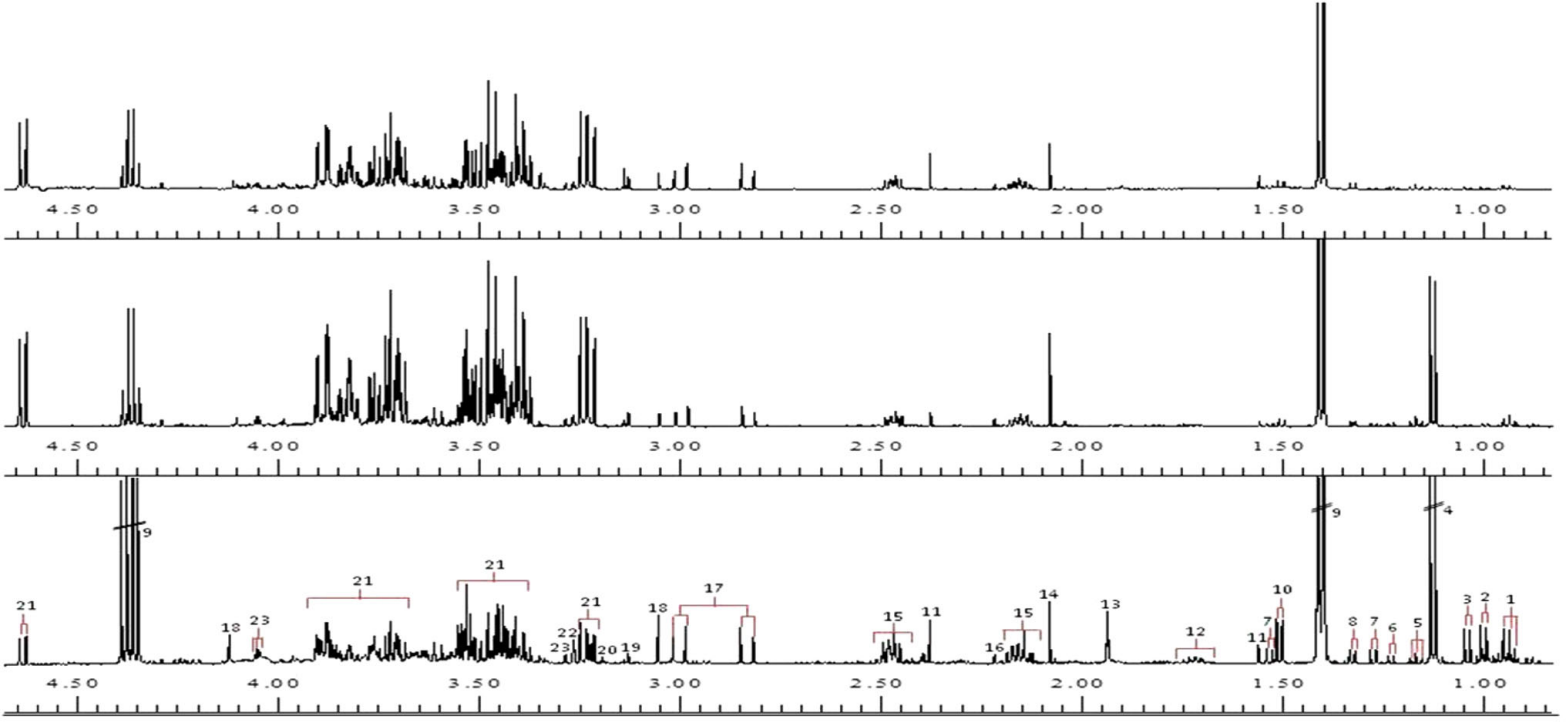
^1^H 500 MHz NMR spectra of CSF at pH 2.5 scaled according to internal standard peak (TSP) illustrating qualitative differences between single examples of untreated NL_Control (*top*), treated SA_Control (*middle*) and TBM cases (*bottom*). [Assignments (ppm): *1* isoleucine/leucine (0.94t/0.95d), *2* valine/isoleucine (1.00d/1.01d), *3* valine (1.04d), *4* propylene glycol (1.13d), *5* ethanol (1.17d, 3.64q), *6* 3-hydroxybutyrate (1.23d, 2.53q), *7* ^13^C lactate (1.30d, 1.52d), *8* threonine/3-hydroxyisovalerate (1.33d/1.33s), *9* lactate(1.41d, 4.36q), *10* alanine (1.51d), *11* pyruvate (1.56s, 2.37s), *12* lysine (1.73 m), *13* unknown, *14* acetate (2.08s), *15* glutamine (2.16m, 2.47m), *16* acetone (2.22s), *17* citrate (2.91AB), 18 creatine (3.05s, 4.10s), *19* creatinine (3.13s, 4.29s), *20* choline (3.19s), *21* glucose (3.23dd, 3.3–3.9, 4.64d, 5.22d), *22* betaine/myo-inositol (3.27s/3.27t), *23* myo-inositol (4.05t)]. Multiplicity of peak(s): *s* singlet, *d* doublet, *dd* double duplet, *t* triplet, *q* quartet, *m* multiplet, *AB* AB system

A representative NMR spectrum, covering the region 0.80–4.80 ppm and scaled relative to the internal standard peak (TSP), from each of the three experimental groups used, is given in Fig. 2. This section of the spectrum serves to illustrate some of the discernible qualitative NMR differences associated with the CSF taken from the TBM-infected infants and small children compared to non-TBM cases. Not shown in Fig. 2 are assignments attributed to medication found within the aromatic region of the spectra (6–9 ppm) from treated individuals, which were identified by comparison with spectra corresponding to the pure compounds, as well as unassigned peaks that have yet to be identified.

**Fig. 3:**
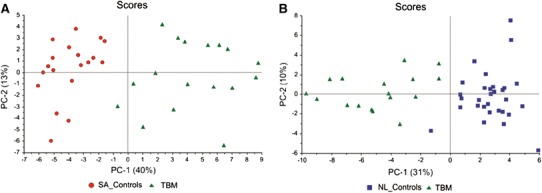
PCA scores plot of SA_Controls vs TBM (**a**) and NL_Controls vs TBM (**b**), showing natural separation between TBM and non-TBM cases

Case and variable reduction was followed as described in Fig. 1. Unsupervised PCA of all 3 experimental groups separated the TBM cases and both control groups, with some overlap between the control groups indicating that they shared similar characteristics but were not homogeneous (see Fig. S1). PCA of the SA_Controls vs TBM (Fig. 3a) and NL_Controls vs TBM (Fig. 3b) cases further illustrates the natural separation between TBM and non-TBM. Quantitatively, the two metabolites that yielded the greatest power values based upon PCA (i.e., those most responsible for the separation) were highly elevated lactate and decreased glucose in the TBM cases relative to normal reference values for CSF glucose (see Table S4).

In order to focus on the biological/metabolic information we subdivided the data matrix into three classes of variables, namely: (1) known endogenous compounds, (2) medications (exogenous), and (3) unassigned variables. To determine the more subtle characteristics that distinguish the host metabolic profile of TBM we selected the known endogenous variables (classifiable as metabolites), which could be confidently assigned given the highly specific nature of NMR technology, and in addition removed the two dominating metabolites—lactate and glucose. This filtering resulted in a reduction of the data matrix under analysis from 109 to 40 variables.

**Fig. 4:**
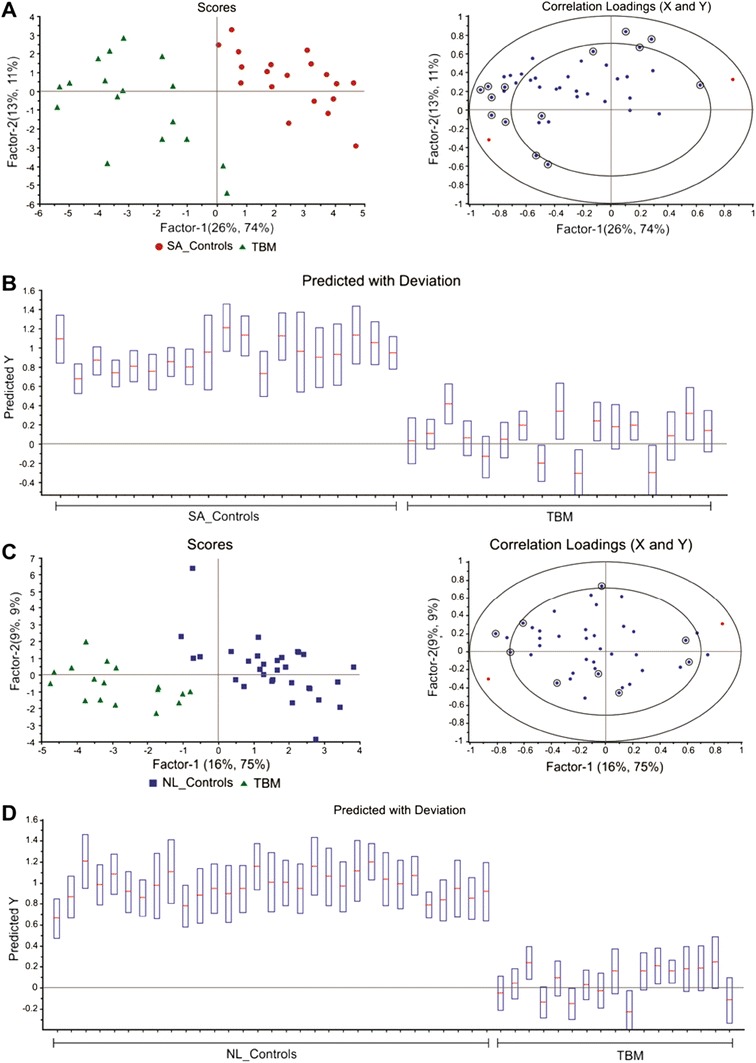
PLS scores plot and corresponding correlation loadings with marked important variables of SA_Control vs TBM (**a**) and NL_Controls vs TBM (**c**) reduced cases. The regression prediction plot illustrates that the PLS model classifies the cases correctly in the SA_Control vs TBM (**b**) and NL_Control vs TBM (**d**) cases

**Fig. 5:**
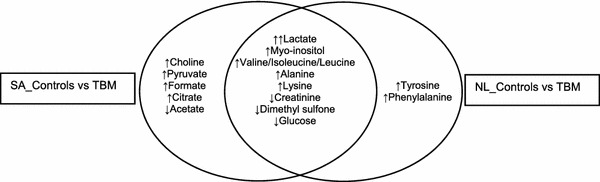
Important metabolites that distinguish between TBM and non-TBM cases based on VIP values > 1.0 and significant univariate measures, common to both cases and unique to each case (increase↑/decrease↓ relevant to TBM cases)

Supervised PLS-DA modelling was performed on the reduced data set for both the SA_Controls vs TBM and NL_Controls vs TBM cases as shown in Fig. 4. The regression model identified the important variables, marked in the corresponding correlation loadings plots, which were then cross-validated and used in a regression prediction with deviation that illustrates—for the SA_Controls vs TBM case (Fig. 4b) and in the NL_Controls vs TBM case (Fig. 4d)—that there is good case classification. The R^2^ and Q^2^ values for the NL_Controls vs TBM case were 0.80 and 0.77 respectively, while the R^2^ and Q^2^ values for the SA_Controls vs TBM case were 0.90 and 0.83, respectively. The fundamental requirement for PLS to yield meaningful information is the ability to select variables. In our case this is done quantitatively on the basis of the VIP value, which, together with other meaningful univariate quantitative measures (fold change d-values and *t* test *p* values), are given in Table 1. Based upon a selection criterion of VIP > 1.0, and taking the univariate measures into consideration, we were able to select 8 significant metabolites common to both cases, namely: myo-inositol, creatinine, dimethyl sulfone and several amino acids [lysine, alanine and three branched-chain amino acids (BCAAs): valine, isoleucine and leucine]. Using the same selection criteria, phenylalanine and tyrosine are uniquely important when comparing the NL_Controls vs TBM case, whereas choline, formate, acetate, citrate and pyruvate are uniquely important to the SA_Controls vs TBM case. Ethanol manifested as important in the NL_Controls vs TBM case but, although very small amounts of ethanol are produced endogenously by bacteria through anaerobic fermentation, it is likely that its presence was due to contamination from skin disinfection prior to lumbar puncture (Fig. 5).

**Table 1:** Quantitative statistical data indicating the important metabolites that discriminate between TBM and non-TBM for both SA_Controls vs TBM and NL_Controls vs TBM cases with dominating metabolites lactate and glucose removed

	PLS	*t* test	Fold change	Validation
	(VIP value)	(*p* value)	(d-value)	(*p* value)
SA_Controls vs TBM				
Choline (3.19)	1.810	<0.001	0.263	<0.001
Alanine (1.51)	1.793	<0.001	0.344	<0.001
Myo-inositol (3.285)	1.708	<0.001	1.605	0.007
Creatinine (3.13)	1.654	<0.001	1.689	<0.001
Lysine (1.73)	1.611	<0.001	0.601	<0.001
Valine/isoleucine (1.01)	1.580	<0.001	0.409	<0.001
Valine (1.04)	1.514	<0.001	0.417	<0.001
Acetate (2.08)	1.371	<0.001	1.635	0.224
Myo-inositol (4.05)	1.338	0.001	1.478	0.366
DMSO_2_ (3.14)	1.110	0.005	1.809	0.072
Formate (8.25)	1.089	0.006	0.651	<0.001
Pyruvate (1.56)	1.083	0.007	0.632	0.001
Citrate (2.81)	1.044	0.009	1.245	0.616
Isoleucine/leucine (0.95)	1.042	0.009	0.715	<0.001
Pyruvate (2.37)	1.011	0.012	0.668	0.005
Tyrosine (7.19)	0.979	0.015	0.688	<0.001
2-Oxoglutarate (2.68)	0.977	0.015	0.511	0.007
Phenylalanine/medication (7.42)	0.947	0.019	0.666	<0.001
Citrate (2.84)	0.944	0.019	1.224	0.838
Threonine (1.34)	0.895	0.027	0.727	<0.001
Creatinine (4.29)	0.891	0.028	1.176	0.185
Creatine (3.05)	0.886	0.029	0.832	<0.001
Phenylalanine (7.39)	0.790	0.053	0.681	0.010
Creatine (4.10)	0.751	0.067	1.230	0.500
Glutamine (2.47)	0.747	0.068	1.098	0.233
Succinate (2.66)	0.699	0.089	0.671	0.016
Acetone (2.22)	0.576	0.164	1.763	0.617
Mannose (5.17)	0.530	0.202	1.626	0.422
Ethanol (3.64)	0.516	0.214	1.145	0.877
Tyrosine/medication (6.90)	0.512	0.218	0.873	0.125
Acetoacetate (2.30)	0.421	0.312	0.951	0.178
Citrate (2.97)	0.367	0.380	1.062	0.327
Glutamine/medication (2.16)	0.287	0.492	1.049	0.090
Ethanol (1.18)	0.237	0.572	1.013	0.491
Betaine/myo-inositol (3.27)	0.202	0.630	0.949	0.002
3-Hydroxybutyrate (2.53)	0.198	0.636	0.999	0.374
3-Hydroxyisovalerate/threonine (1.33)	0.113	0.789	0.949	0.003
Citrate (3.00)	0.083	0.843	0.995	0.002
Carnitine (3.22)	0.031	0.940	0.952	0.026
3-Hydroxybutyrate (1.23)	0.018	0.965	1.123	0.882
NL_Controls vs TBM				
Alanine (1.51)	1.986	<0.001	0.299	<0.001
Valine (1.04)	1.961	<0.001	0.320	<0.001
Creatinine (3.13)	1.938	<0.001	1.860	<0.001
DMSO_2_ (3.14)	1.851	<0.001	3.132	<0.001
Myo-inositol (3.285)	1.836	<0.001	1.778	<0.001
Creatinine (4.29)	1.557	<0.001	1.705	<0.001
Myo-inositol (4.05)	1.497	<0.001	1.734	0.003
Valine/isoleucine (1.01)	1.477	<0.001	0.449	<0.001
Lysine (1.73)	1.441	<0.001	0.592	<0.001
Ethanol (1.18)	1.329	0.001	0.314	0.067
Tyrosine/medication (6.90)	1.274	0.002	0.620	0.002
Phenylalanine/medication (7.42)	1.092	0.009	0.698	<0.001
Tyrosine (7.19)	1.085	0.009	0.726	<0.001
Phenylalanine (7.39)	1.074	0.010	0.578	0.003
Acetoacetate (2.30)	0.825	0.051	1.130	0.164
Choline (3.19)	0.801	0.058	0.770	0.125
Isoleucine/leucine (0.95)	0.788	0.063	0.804	0.005
Citrate (2.84)	0.730	0.085	1.284	0.121
Creatine (4.10)	0.693	0.103	1.222	0.269
Pyruvate (1.56)	0.649	0.128	0.858	0.026
Pyruvate (2.37)	0.642	0.132	0.845	0.042
Glutamine (2.47)	0.638	0.134	1.131	0.428
Mannose (5.17)	0.569	0.183	4.641	0.075
Creatine (3.05)	0.540	0.207	0.907	0.295
3-Hydroxybutyrate (2.53)	0.431	0.315	1.019	0.794
Acetate (2.08)	0.382	0.374	1.135	0.501
Betaine/myo-inositol (3.27)	0.348	0.418	1.104	0.353
3-Hydroxyisovalerate/threonine (1.33)	0.330	0.442	1.179	0.715
Ethanol (3.64)	0.306	0.477	1.074	0.513
Glutamine/medication (2.16)	0.221	0.607	0.977	0.419
Formate (8.25)	0.209	0.627	0.967	0.440
Carnitine (3.22)	0.206	0.633	1.043	0.857
Citrate (3.00)	0.203	0.637	1.032	0.362
Citrate (2.97)	0.197	0.647	1.025	0.510
Threonine (1.34)	0.183	0.671	1.080	0.672
Acetone (2.22)	0.172	0.689	1.681	0.752
Citrate (2.81)	0.164	0.703	1.064	0.685
3-Hydroxybutyrate (1.23)	0.149	0.730	1.364	0.722
Succinate (2.66)	0.133	0.758	1.103	0.640
2-Oxoglutarate (2.68)	0.111	0.797	1.060	0.456

The chemical shift, in ppm, of each identified metabolite is given in brackets

Thus a total of 16 NMR-derived CSF metabolites, which differentiated between the South African TBM cases under investigation and controls, were selected for quantification in the 17 TBM, 19 SA_Control and 30 NL_Control cases and the outcome compared to their normal reference ranges for human CSF (summarized output presented in Table 2).

**Table 2:** Summarized quantified data of 16 important metabolites discriminating between TBM and controls, compared with normal reference ranges

Metabolite*		Isoleucine/Leucine	Valine	Alanine	Lysine	Phenylalanine	Tyrosine	Creatinine	Acetate	Pyruvate	Citrate	DMSO_2_	Choline	Myo-inositol	Lactate	Glucose^c^
Chemical form (shift)		(CH_3_)_2_ (0.95t)	CH_3_ (1.00d)	CH_3_ (1.51d)	CH_2_ (1.73m)	(CH)_3_ (7.35m)	(CH)_2_ (7.19d)	CH_3_ (3.13s)	CH_3_ (2.08s)	CH_3_ (2.36s)	CH_2_ (2.89AB)	(CH_3_)_2_ (3.14s)	(CH_3_)_3_ (3.19s)	(CH)_2_ (4.05t)	CH_3_ (1.41d)	α-CH (5.22d)
NL_Controls	Mean	18.18	12.69	23.20	24.75	4.74	5.62	52.34	120.15	45.70	135.30	10.70	2.11	201.36	1,394.81	2,838.15
	STD	9.31	5.75	12.43	13.65	5.02	5.15	24.02	78.60	30.13	48.00	6.44	1.34	203.23	619.61	933.18
NL_Controls vs TBM *t* test (*p* value)		<0.001	<0.001	<0.001	<0.001	<0.001	<0.001	0.506	0.028	<0.001	<0.001	0.003	0.002	0.306	<0.001	0.640
SA_Controls	Mean	26.44	16.06	37.28	29.52	6.11	13.60	58.96	192.98	36.00	172.90	7.75	2.10	169.85	1,706.83	4,561.79
	STD	11.01	9.63	13.52	13.44	9.18	19.08	16.42	50.30	26.53	45.16	4.72	1.42	47.52	419.06	1,101.83
SA_Controls vs TBM *t* test (*p* value)		<0.001	<0.001	0.028	<0.001	<0.001	0.005	0.075	0.736	<0.001	0.015	0.094	0.013	0.400	<0.001	<0.001
TBM	Mean	54.01	69.49	99.47	86.60	37.44	31.04	47.70	183.38	82.03	206.02	4.86	4.34	177.30	7,363.69	2,689.05
	STD	27.55	40.05	71.92	40.72	18.44	14.81	20.29	111.22	35.60	29.47	5.35	3.39	95.59	2,361.32	1,216.99
Reference range		7 ± 5/16 ± 9^a^	19 ± 13^a^	46 ± 27^a^	29 ± 13^a^	15 ± 13^a^	12 ± 9^a^	43 ± 12^a^	58 ± 27^a^	53 ± 42^a^	225 ± 96^a^	2 ± 1^a^	3 ± 1^a^	84 ± 40^a^	1,651±626^b^	2,960 ± 111^b^

* Concentration units of metabolites are µmol/L

^a^Wishart et al. 2008

^b^Leen et al. 2012

^c^Total glucose concentration calculated based on α-glucose concentration and using α:β ratio of 39:61 (Boss et al. 2000)

### Brain energy metabolism and the response to TBM

The clear and defining metabolites distinguishing the TBM cases under investigation from the control groups were the combination of perturbed glucose and highly elevated lactate, which is in line with the clinical characteristics of CSF from patients with BM and TBM (Leib et al. 1999) and is generally accepted as a key indication of disturbances in energy metabolism of many neurological disorders (Leen et al. 2012). Regional variation and the complement of cellular determinants participating in a synchronised way is a unique characteristic of neurological energy production. It has thus been proposed that the microvascular endothelium of the blood–brain barrier (BBB), astrocytes, neurons and extracellular matrix constitute a “neurovascular unit”, which is essential to normal CNS physiology and health (reviewed by Hawkins and Davis 2005).

A basic functional component in this unit is the metabolic astrocyte–neuron coupling. The astrocytes participate through glutamate-stimulated aerobic glycolysis, resulting in the release of lactate, thereby serving as fuelling in conditions of an activity-dependent neuronal energy demand (Magistretti and Pellerin 1999). Several experiment-based insights (reviewed by Pellerin and Magistretti 2003 and Pellerin and Magistretti 2004) lend empirical support to the original proposed “astrocyte–neuron lactate shuttle” model on energy transfer in the CNS (Bittar et al. 1996; Pellerin et al. 1998). Recent views highlight the importance of brain endothelial cells in the modular organization of the neurovascular unit (Abbott et al. 2006) and summarize how several pathologies of the CNS may be involved in the disturbance of this organization, including pathological conditions like TBM. The resulting profile of energy-associated metabolites in CSF from TBM patients (Table 1) differs significantly from profiles of the metabolites associated with the controls in several ways.

#### Metabolic burst

The increased catabolism of glucose through glycolysis to pyruvate, and its conversion to alanine and predominantly lactate, reflects a metabolic burst as seen in the TBM CSF profile, with the greatly elevated levels of lactate, most likely leading to immune activation in the TB-infected microglial cells (Nareika et al. 2005). In addition, the increased levels of many monocarboxylates such as lactate and pyruvate, the presence of branched-chain keto acids derived from leucine, valine and isoleucine, and ketone bodies such as acetoacetate, 3-hydroxybutyrate and acetate suggests functional down regulation of the neuron-associated monocarboxylate transporter-1 (MCT-1), resulting in decreased neuronal energy supply, which manifests in the coma-related clinical symptoms seen in the TBM patients. Down regulation of neuronal MCT-1 likewise promotes increased levels of these monocarboxylic acids and ketones, which could be destined for high-energy support to activated microglia operative in TBM. Some pyruvate is converted to alanine by a transaminase reaction. The ammonia necessary for this reaction depends on the presence of branched amino acids, leading to an increase in the branched-chain 2-keto acids. It should be noted that acetyl-CoA is an important precursor to the tricarboxylic acid cycle (TCA) for energy production, not only in the host, but is also one of the primary sources of energy for Mtb (Savvi et al. 2008). In Mtb, formate is a by-product of acetate production and may inhibit downstream mitochondrial energy production in the host, limiting macrophage activation.

#### Amino acids

The (BCAAs—isoleucine, leucine and valine) participate directly and indirectly in a variety of important biochemical functions in the brain, including the provision of building blocks for the production of energy through gluconeogenesis under conditions of high-ATP demands as prevails in activated microglia. BCAAs and alanine also participate in intercellular shuttles between mitochondria in astroglia and neurons (Sweatt et al. 2003). Likewise, a basic aspect of neurometabolic coupling is the glutamate/glutamine link in the glycolytic catabolism in astrocytes, lactate production and its availability to mitochondrial ATP production in neurons, or proposed for microglia in TBM. Increased lysine seen in CSF from TBM patients has been associated with mental retardation and in other motor neuron diseases (Shaw et al. 1995); it has also been shown to form adducts with other compounds, such as acrolein–lysine, a marker of lipid peroxidation in childhood meningitis (Tsukahara et al. 2002).

#### Creatinine

A catabolic product of creatine phosphate in muscle that is produced at a fairly constant rate during homeostasis and has a slow diffusion rate across the BBB. It is also correlated with the CSF monoamine metabolites homovanillic acid and 5-hydroxyindoleacetic acid, which are implicated in neurodegenerative conditions such as depression and schizophrenia (Agren and Niklasson 1988; Levine et al. 2000; Swahn and Sedvall 1988). Implications of perturbed creatinine in TBM is unclear but are likely linked proportionally to the progression of neuronal injury.

#### Myo-inositol

This carbohydrate is synthesized mainly *de novo* from glucose in the brain. As phosphoinositides, they participate as second messengers in numerous neurotransmitter systems and are important in signal transduction (Cordoba et al. 1996). Myo-inositol is a glial cell marker that has been implicated previously in activation of microglia and astrocytes (gliosis), as well as a known pathological response readily observed in neurodegenerative disease and neuroinflammation (Pears et al. 2005). Thus myo-inositol is known to be crucial in the initiation of the cascade of events necessary for an immune response, although the mean concentrations found in the present study do not strongly highlight this function of myo-inositol.

#### Choline

Choline is synthesized in small amounts in the brain by converting phosphatidylethanolamine to phosphatidylcholine—although its main source is dietary—and can be oxidized to form betaine, which is a methyl source for many reactions (e.g., conversion of homocysteine to methionine). It is an important precursor of the neurotransmitter acetylcholine, which activates muscles in the peripheral nervous system and, in excess, induces seizures (Zimmerman et al. 2008), a common clinical symptom in TBM. Increased choline can be related to neural membrane breakdown, reflecting neuronal loss and gliosis in the brain.

#### Dimethyl sulfone (DMSO_2_)

DMSO_2_ is a normal constituent of human CSF, occurring in concentrations of 0–25 μmol/L and is derived from dietary sources, intestinal bacterial metabolism and human endogenous methanethiol metabolism. Metabolically, DMSO_2_ arises from oxidation of dimethyl sulfoxide (DMSO), and partly from methionine metabolism (Engelke et al. 2005). DMSO is a polar amphiphilic compound that increases membrane fluidity (Hallows and Frank 1992), acts as a powerful scavenger of oxygen radicals (Rosenblum 1983), stimulates granulocytic differentiation (Watson et al. 1997) and induces apoptosis of neuroblastoma cells (Kruman et al. 1993) and macrophages (Marthyn et al. 1998). Decreased levels of DMSO_2_ in CSF can thus be postulated as being a consequence of perturbed osmoregulation of CSF and depletion of DMSO in response to oxidative stress, induced apoptosis and differentiation of macrophages.

### A hypothetical “astrocyte–microglia lactate shuttle” (AMLS)

The pathophysiological response to neural infection by Mtb provided the context for our interpretation of the resultant metabolic changes outlined above. Clinical observations in our TBM patient group include the classical profile of encephalopathy with manifestations of seizures, involuntary movements, fever, impaired consciousness, poor feeding and vomiting, indicative of neuroinflammatory responses and impaired neuronal functioning (Udani and Dastur 1970). Concomitantly, microglial cells are likely to pose an increased energy demand for phagocytosis of Mtb, most likely strengthened by anaerobic glycolysis in the activated microglia, although this response appears to be insufficient as the energy provider for an inflammatory response (Voloboueva et al. 2013). A present paradigm is that lactate functions as a key intermediate in conditions of increased energy demand, based on the notion that glycolytic and oxidative pathways can also be operationally linked, as opposed to alternative processes only. Lactate, the product of the anaerobic pathway, thus becomes the substrate for the aerobic pathway—reminiscent of the classical observation by Carl and Gerty Cori (Cori and Cori 1928) on the conversion of muscle glycogen to liver glycogen with lactic acid as an intermediary stage, now commonly known as the Cori-cycle. The intermediary role of lactate led to a postulated lactate shuttle (Brooks 1986), even prior to the discoveries of several classes of transport factors had been implicated in this process in the CNS (See “Effector isoforms” as discussed in section S4 of the SI). Various proposals exist on the involvement of lactate in the astrocyte’s role in cell–cell participation under neurostress conditions, enabled also through the syncytial network of neuronal cells within the expansive parenchymal domain of the CNS:

1. Initial in vitro experiments indicated that lactate is an efficient energy substrate for neurons, particularly during periods of intense activity. Lactate does not cross the BBB easily, which excludes the possibility that blood-borne lactate can be a primary source for this energy provision, although several investigations indicate that astrocytes could release large amounts of lactate, leading to the hypothesis of an activity-dependent astrocyte-neuron lactate shuttle (ANLS) for the supply of energy substrates to neurons (Pellerin et al. 1998). Although the ANLS hypothesis provides an important existing paradigm on energy provision to neurons, alternative views on activated (Patel et al. 2014) as well as resting neurons (reviewed by Dienel and McKenna 2014) also strongly prevails, and provides considerable scope for alternative investigations on hypothesis testing.
2. Lactate is produced continuously under fully aerobic conditions in mammalian skeletal muscle, especially during exercise when rates of glycogenolysis and glycolysis are elevated, with active skeletal muscles being capable of lactate removal, mainly via oxidation, from which an intracellular lactate shuttle (ILS) has been postulated (Brooks 2002). Investigations with primary cultures from rat cortex and hippocampus, including immunohistochemistry and immunoprecipitation techniques, indicated that monocarboxylate transporter-2 and lactate dehydrogenase are co-expressed in the mitochondria of cultured neurons, which gave support to the concept that in neurons, as in skeletal muscle, a mitochondrial ILS could be operative (Hashimoto et al. 2008). It has thus been proposed that lactate seems to play a critical role in neuronal survival, memory and conditions like Alzheimer’s disease, although more functional evidence is needed to confirm this hypothesis (Newington et al. 2013).
3. Most recently, a microglia–astrocyte–neuron lactate shuttle has been proposed for microglial activation with lipopolysaccharide (LPS) and interferon-γ, as well as in response to conditions of excitotoxicity. Both conditions imply the participation of a four-component constellation in which lactate is shuttling to neurons not only from astrocytes but also from microglia (Gimeno‐Bayón et al. 2014). In this model, glucose, oxidized by astrocytes and microglia, are converted into lactate that is taken up by neurons for its complete oxidation, while glutamine and glutamate synaptic removal by microglia fuels the TCA cycle to maintain mitochondrial activity and ATP generation.

Finally, lactate not only serves as a fuel source and gluconeogenic precursor, but it also acts as a signalling molecule (Philp et al. 2005; Hashimoto et al. 2007). Hashimoto and co-workers demonstrated that lactate increased production of reactive oxygen species (ROS) and up-regulated 673 genes, many known to be responsive to ROS, in L6 myogenic cells. It thus appears that increased lactate augments inflammatory gene expression.

Against this multi-paradigm background, we speculated that the inflammatory responses and metabolic imbalances created in the CNS following the initial phases of infection by a pathogen, such as Mtb, should be to the advantage of the microglia to fulfil their immune-protective function. For this purpose we advanced the following hypothesis: ‘The host’s response to neural infection results in an AMLS that operates in neuroinflammatory diseases, such as TBM.’ We formulated this hypothesis on the metabolite information on TBM in children and infants (summarized in Table 2), with special reference to the significant increase in lactate in all the TBM cases, as well as through inductive reasoning (Goodacre et al. 2004) on the characteristics of the cell–cell interactions and factor isoforms, which are well-established to be operative in normal and stress-induced conditions in the CNS (see detailed discussion in section S4 of the SI).

Briefly, it is postulated that in TBM, lactate produced through glycolysis in astrocytes participates in the activated immune response and, in association with ketones and gluconeogenic amino acids, is collectively directed from the neurons preferentially into microglia where it enters the mitochondrial TCA cycle, contributing to oxidative phosphorylation and hence producing high levels of ATP and forms of ROS required for Mtb degradation. ROS, and a multitude of factors produced by the microglia to modulate the functions of surrounding immune cells, are toxic to neurons and the unregulated activation of microglia in response to stimulants, such as Mtb, propagate neuronal injury (Block and Hong 2005) and eventual apoptotic cell death for the over-activated microglia as well (Liu et al. 2001).

Activation of microglia, as implied by the AMLS hypothesis, does not, however, present a uniform process and involves intricate interactions and feedback loops between the microglia, astrocytes and neurons that hamper attempts to construct basic and linear cascades of cause and effect; TBM involves a complex integration of the responses from the various cell types present within the CNS, with microglia and the astrocytes as main players. The initiating result is the infiltration of Mtb into the subarachnoid space followed by immune activation, but several other factors, related to age, genetic background as well as societal factors from the environment and past experiences, are all expected to modulate the integrated response of this complex neuroinflammatory pathology underlying TBM. We also propose a conceptual model, encapsulating the AMLS hypothesis and providing a visual representative of theoretical constructs (see Fig. S2), as further exploration of the pathophysiological responses in TBM aimed at fostering curiosity and stimulating new ideas in research on this serious disease.

## Concluding remarks

The results from the metabolomics analysis of the CSF from TBM-infected small children, the metabolite profile of the host response from which it is derived, and the avenues opened by the AMLS hypothesis, provide for further reflection and stimulus, especially for improvement of early clinical assessment of TBM, timely introduction of treatment and for monitoring responses to applicable treatment regimes. Finally, despite its declining incidence in most high-income countries, tuberculosis shows no signs of disappearing in the near future and prevails as a major burden of disease affecting high-risk groups, such as socio-economically deprived individuals and especially infants and children. This investigation indicates that a metabolomics analysis of TBM is feasible and a potentially important complementary tool in combating this devastating disease.

## Electronic supplementary material

Below is the link to the electronic supplementary material.

Supplementary material 1 (DOCX 5432 kb)
