# Supplementary material for: A hypothetical astrocyte–microglia lactate shuttle derived from a 1H NMR metabolomics analysis of cerebrospinal fluid from a cohort of South African children with tuberculous meningitis
Source: Metabolomics. 2014 Oct 11;11(4):822–37. doi: 10.1007/s11306-014-0741-z (PMC4475545; doi:10.1007/s11306-014-0741-z)
Supplement: Supplementary file 1 — Supplementary material 1 (DOCX 5432 kb) [file 11306_2014_741_MOESM1_ESM.docx]

**Supplementary Information**

**A hypothetical astrocyte**–**microglia lactate shuttle derived from a ^1^H NMR metabolomics analysis of cerebrospinal fluid from a cohort of South African children with tuberculous meningitis**

Shayne Mason^1^, A. Marceline van Furth^2^, Lodewyk J. Mienie^3^, Udo F.H. Engelke^4^, Ron A. Wevers^4^, Regan Solomons^5^, Carolus J. Reinecke^1^

^1^Centre for Human Metabonomics, Faculty of Natural Sciences, Private Bag X6001, North-West University (Potchefstroom Campus), South Africa,

^2^Department of Paediatric Infectious Diseases–Immunology and Rheumatology, Vrije Universiteit Medical Centre, De Boelelaan 1117, 1081 HV Amsterdam, The Netherlands,

^3^Potchefstroom Laboratory for Inborn Errors of Metabolism, School for Biochemistry, Private Bag X6001, North-West University (Potchefstroom Campus), South Africa,

^4^Radboud University Nijmegen Medical Centre, Department of Laboratory Medicine, PO Box 9101, 6500 HB Nijmegen, The Netherlands,

^5^Department of Paediatrics and Child Health, Tygerberg Hospital, University of Stellenbosch, PO Box 19063, Tygerberg 7505, South Africa.

**Contents**

**S1 Sample and clinical information on patients and controls**

**S2 Experimental aspects of the NMR protocol**

**S3 Data preprocessing**

**S4 Conceptual model of the AMLS hypothesis**

**S5 Tables and figures**

**Table S1**: General clinical symptoms of TBM cases in duration (days) prior to admission to hospital, evidence of TB outside the CNS and stage of TBM disease.

**Table S2**: Clinical chemistry of CSF describing elevated immuno-cells, decreased glucose and elevated proteins in TBM cases compared to SA_Control cases.

**Table S3**: Summary of total, and type of, medication administered to the TBM and SA_Control cases analysed in this study prior to admission to hospital.

**Table S4**: PCA power values of SA_Controls vs TBM and NL_Controls vs TBM cases for all 109 variables, with a cutoff value of 0.5, illustrating quantitatively that lactate and glucose were the two dominant discriminating metabolites.

**Table S5**: Quantitative data for all identified variables indicating the most important metabolites in discriminating between TBM and non-TBM for both SA_Controls vs TBM and NL_Controls vs TBM cases; which includes: PLS-DA VIP values, fold change d-values and p-values for the t-test and from the cross-validation of the PLS model.

**Figure S1**: PCA scores plot showing natural separation between TBM and non-TBM cases, with overlapping between control groups, and associated loadings plot for all 109 variables.

**Figure S2**: Conceptual model of the AMLS hypothesis

**S1 Sampling and clinical information on patients and controls**

The three experimental groups used in this study were: 1) South African patients with confirmed TB meningitis, 2) non-meningitis South African controls (SA_Controls), and 3) neurological controls from the Netherlands (NL_Controls). The first two groups comprised children between the ages of 6 months and 12 years, all of whom were originally suspected meningitis cases and referred from local clinics to the paediatrics unit at Tygerberg Hospital in the Western Cape province of South Africa. In most of these cases, a broad range of non-specific treatments (e.g., broad-spectrum antibiotics, analgesics and anti-inflammatories) were initiated prior to admission. These individuals were given a thorough assessment by a paediatric neurologist at Tygerberg Hospital, which involved an extensive description of the clinical background, including analysis of a CSF sample obtained through a lumbar puncture. The main physical presentations and clinical symptoms of these cases, as testified by parents and observed by the respective clinicians (see Table S1), were compatible with non-CNS indications of TB outside the CNS, typically observed in small TB-infected children from the region. As part of the diagnostic process a detailed inspection of the clinical chemistry of the CSF was conducted (see Table S2) and yielded a report describing the count and type of cells present, particularly immune response-related cells, as well as a measure of CSF protein (mostly high: >1 g/L) and glucose (mostly low: <2.2 mmol/L or CSF:bBlood glucose ratio <50%) levels.. Most, but not all, of the children received a wide range of medication uniquely based upon their presentation of clinical symptoms, which included: TB-specific drugs, antibiotics and analgesics, anti-inflammatory drugs, sedatives and drugs directed to specific symptoms like seizures or for directed treatment (e.g. diuretics, laxatives, muscle relaxants, anti-psychotics), as well as multivitamins and other supplements summarized in Table S3.

Approximately 1 mL of the CSF samples was stored at –80^o^C for this metabolomics study, for which informed consent from the respective parents was obtained. Following polymerase chain reaction (PCR), clinical chemistry and culture analysis of the CSF, an informed diagnosis was made where those in the patient group were confirmed positive for TB meningitis. The South African controls were confirmed negative for any form of meningitis, despite being ill with symptoms indicative of meningitis. Ethical and practical considerations limit the availability of obtaining healthy controls, which could partially be overcome through a comparative analysis of a second control group involving CSF collected from untreated individuals from the Radboud University Medical Centre in Nijmegen, the Netherlands. The Netherlands non-TBM control group (the third experimental group) consists of CSF samples from age-matched patients who were suspected to suffer from a neurometabolic disease. After appropriate and in depth investigations no clinical or biochemical evidence was found for such diagnosis in any of these patients. The only exclusion criterion applied to all cases was HIV co-infection.

**S2 Experimental aspects of the NMR protocol**

All CSF samples were stored at –80°C prior to analysis and transported to the NMR facility at the Laboratory for Genetic, Endocrine and Metabolic Diseases at the Radboud University Medical Centre in Nijmegen, the Netherlands, where the NMR analyses were done. Sample preparation followed the standard operating procedure (SOP) as set up by the Nijmegen laboratory (Engelke *et al.* 2005; Engelke *et al.* 2006; Wevers *et al.* 1995). The first step involved cleaning the Sartorius Centrisart^®^1 10-kDa centrifugal unit by pre-centrifugation twice (washing involved centrifugation with distilled water for 10 min at 3000 rpm in order to remove excess glycerol in the filter membrane), followed by centrifugation of approximately 1 mL CSF sample for 15 min at 3000 rpm; 700 µL ultrafiltrate was collected (in those cases where there was insufficient volume the ultrafiltrate was diluted to 700 µL final volume with distilled water and the dilution factor noted) and 20 µL internal standard (20.2 mM trimethylsilyl-2,2,3,3-tetradeuteropropionic acid (TSP, sodium salt; Aldrich) in deuterium oxide) added, after which the pH was adjusted to 2.50 ± 0.05 using concentrated HCl. Finally, 650 µL of the prepared sample was analyzed in a 5-mm NMR tube.

Each CSF sample was measured at 500 MHz on a Bruker DRX Avance spectrometer equipped with a triple-resonance inverse (TXI) ^1^H{^15^N, ^13^C} probe head and equipped with x, y, z gradient coils. ^1^H spectra were acquired as 128 transients in 32k data points with a spectral width of 6002 Hz. The sample temperature was 298 K and the H_2_O resonance was pre-saturated by single-frequency irradiation during a relaxation delay of 10 s, and a 90° excitation was used. Shimming of the sample was performed automatically on the deuterium signal. The resonance line widths for TSP and metabolites were <1 Hz. A π/2-shifted sine-bell window function was applied to the free induction decay. Fourier transformation was performed after zero-filling to 64k data points. The phase and the baseline were corrected manually. Software used was: Bruker Topspin(V3.1) for data pre-processing and Bruker AMIX(V3.9.12) for binning and quantification (Ellinger *et al.* 2013). A final bucket data matrix was created scaled relative to the total spectrum intensity and final metabolite concentrations quantified as µmol/L, using the TSP peak as a reference. A Bruker spectral library database and an in-house pure compound database were used to identify NMR peaks.

**S3 Data pre-processing**

Some of the variation that can occur in NMR spectra includes: spectral noise, changes in chemical shifts and peak widths due to fluctuations in pH, instrument and/or temperature. This variation can be minimized by spectral binning, which is a common approach in NMR-based metabolomics (Wishart 2008) and involves dividing the NMR spectra into regions, also known as frequency windows, or ‘buckets’, of defined width. The norm with NMR-based metabolomics studies involves segmenting the NMR spectrum into set width rectangular buckets (e.g., 0.005 ppm bin width (Viant *et al.* 2005; Lin *et al.* 2007; Sinclair *et al.* 2009), 0.04 ppm bin width (Pears *et al.* 2005; Waters *et al.* 2001), and 0.02 ppm bin width (Pan *et al.* 2007)). The total cumulative peak intensity of these buckets is summed and each bucket is then represented as a fraction relative to this total, effectively smoothing (normalizing) the spectrum. While this smoothing serves to stabilize and reduce the variation between spectra, accounting for random variation, dilution and bulk mass differences, it also reduces resolution and can introduce other errors through the rigid and inaccurate definition of the bin boundaries that can produce artefacts (Craig *et al.* 2006; Ellinger *et al.* 2013; Powers 2009). To overcome the possibility of inducing such error, variable-sized bucketing was used in this study which, based upon prior spectral knowledge of typical CSF profiles and an understanding of the clinical information associated with the experimental cases, utilized user-defined bin widths that are unique and specific to the data set examined here. Based upon this customized spectral binning, each spectrum was divided into 110 buckets, each associated with one specific chemical substance (variable), unless overlap with more than one substance occurs within a specific bucket. These data were transposed into an *n* x *m* matrix, where *n* is the number of cases (rows) and *m* is the number of variables (columns) – in our case *m* =110 – and subsequently log-transformed. Log transformation of the binned data causes the variance of the spectral intensity in each bin to become more constant, allowing for more efficient multivariate analyses (Purohit *et al.* 2004; Lin *et al.* 2007; Viant *et al.* 2005). In the case of untransformed data, the bins with the highest spectral intensity (largest variance) will dominate the analysis. The NMR region representing the suppressed water signal (4.67–4.96 ppm) was excluded from the data matrix.

**S4 A conceptual model of the AMLS hypothesis**

*Conceptual modelling*

Model development provides a way of integrating biological insights, and of combining the interpretation of experimental results and existing views in the literature into functional contexts, resulting in a wide array of possible models [e.g., conceptual, mathematical, computational, and informational], depending on the purpose of the modelling initiative (Goel *et al*. 2006). In their review, Goel *et al*. emphasize that modelling often requires that purely descriptive approaches to biology be accompanied by the ability to make reliable, quantitative predictions of the responses of cells or organisms to experimentally untested situations. In this SI we present a conceptual model on Mtb for discussion purposes. Conceptual models differ from experiment- and data-based models as they are formed by a mental process, resulting in visual representations which are constructed to facilitate understanding. They incorporate diverse inputs that can relate to each other through a common framework and have predictive value to promote new views or hypotheses. Our conceptual model of the AMLS hypothesis, formulated for TBM, is based on the interpretation of the clinical profile of the TBM and control cases, the outcomes of the NMR metabolomics investigation, as well as on an interpretation of CNS metabolism, which we derived from contemporary knowledge and paradigms prevailing in the scholarly literature. Central nervous system (CNS) metabolism and regulation cover a vast field, and for the present purpose we only focused on: (1) astrocyte and microglia activation in response to TB infection of the meninges; (2) cell–cell communication; (3) metabolic coupling, and (4) effector isoforms.

*Astrocyte and microglia activation*

Astrocytes and microglia act as immune surveillant cells of the nervous system to protect the integrity of the CNS. Under normal physiological conditions, astrocytes buffer ions and biogenic amines provide trophic and survival factors for neurons and oligodendrocytes, stabilizing and maintaining CNS homeostasis. Likewise, microglia produce neuro-specific factors that are important in neuronal survival and thus concomitantly contribute to homeostasis and neuroplasticity in the brain (Merrill and Jonakait 1995). During endogenous or exogenous stress conditions in the CNS – induced, for example, by brain lesions (stroke, head injury), chronic diseases (multiple sclerosis, Alzheimer’s or Parkinson’s diseases) or infectious states (viral, bacterial or, specifically, tuberculous meningitis) – microglia and astrocytes become activated and de-regulated relative to the homeostatic state (Chen and Swanson 2003). The reaction of astrocytes to brain injury is similar in some respects to the inflammatory response of peripheral tissues. Inflammatory activation results in the multistep process of leukocyte invasion in the brain, with leukocytes, in particular the presence of lymphocytes in the CSF, being the diagnostic hallmark of meningitis, as seen in TBM patients in the present study (Table S2). Simultaneously, microglial cells function as the resident macrophages of the brain parenchyma; they also share many, if not all, of the properties of macrophages in other tissues (Nareika *et al.* 2005), such as production of a variety of cytokines and chemokines on activation. In addition, it has become progressively clear that astrocytes and microglia, in conjunction with neurons and endothelial cells of microvessels, are organized into well-structured neurovascular units (Abbott *et al.* 2006), which are involved in the regulation of cerebral blood flow and CNS homeostasis. As homeostatic signalling within the neurovascular unit underlies normal brain function, we regard it as conceptually sound to postulate that perturbed signalling and reordered metabolic functioning might become operational under conditions where stressors, such as pathogenic organisms, invade the CNS. This led us to the formulation of the AMLS hypothesis as outlined in the main text.

The model shown in Fig. S2 aims to address some conceptual aspects in relation to this hypothesis. We recognize that the complex CNS metabolism and distinct regulatory metabolic pathways exclude incorporation of important mechanistic features in the model. We nonetheless attempted to integrate several conceptual paradigms and our own metabolomics information into the conceptual framework. We believe that the diverse inputs sufficiently relate to each other to stimulate new views on TBM, critical assessment of the AMLS hypothesis and, we hope, pave the way for its experimental validation or improvement.

*Cell–cell communication*

Non-neuronal glial cells (e.g., astrocytes, microglia and oligodendrons) have unique cellular structures and phenotypic characteristics to sense their surroundings and respond dynamically to changes in their microenvironment (reviewed by Allaman *et al.* 2011). They constitute the macrophage complement of the CNS; the microglial uptake of Mtb is facilitated by receptor mediation (Peterson *et al.* 1995). Their subsequent transformation to a reactive state with a high energy demand, known as microglial activation, includes morphological changes and appears to produce diverse and dynamic transcriptional and non-transcriptional responses, which have clear functional consequences for the communication network between glial cells and neurons (Allaman *et al.* 2011) through direct membrane contact, soluble signalling mediators or exosomes.

*Metabolic coupling*

Increased physiological activation of neural functions is accompanied by additional energy demand, provided through glucose utilization in metabolic coupling between astrocytes and neurons (Pellerin and Magistretti 1994). This coupling became expressed in a hypothetical “astrocyte–neuron lactate shuttle” (Pellerin *et al.* 1998), where the astrocytes perform glycolysis under aerobic conditions, export the metabolic end-products (pyruvate and lactate) for uptake by neurons, and stimulate the neuronal TCA cycle and oxidative phosphorylation to generate large amounts of ATP. Conceptually, the “Reverse Warburg Effect” (Pavlides *et al.* 2009), proposed as operating in tumour cells, has been interpreted to be analogous to the astrocyte–neuron metabolic coupling (Pavlides *et al.* 2010), where the astrocytes resemble cancer‐associated fibroblasts and the neuron the epithelial tumour cells, resulting in their higher proliferative capacity.

*Effector isoforms*

A wide array of isoenzymes and transporters (chaperones) participate in and affect neural metabolism. They have a crucial role as catalysts and gatekeepers of the metabolic bidirectional cell–cell interactions. Their respective catalytic capacity (isoenzymes), or their up- or down-regulation (transcriptional or otherwise), have an important role in homeostasis or under conditions of increased physiological activation of specific neural functions. (1) The pyruvate dehydrogenase complex (PDC) functions as the rate-limiting point of entry for pyruvate into the TCA; the phosphorylation status of the pyruvate dehydrogenase alpha (PDHα) subunit of the PDC has been shown to distinguish the metabolic phenotypes between astrocytes and neurons (Halim *et al.* 2010; Xing *et al.* 2012). (2) Five lactate dehydrogenase (LDH) isoenzymes catalyze the interconversion of lactate and pyruvate – with LDH-l (neuron located) being inhibited by pyruvate – and preferentially drive the reaction towards the production of pyruvate. The LDH-5 subunit (astrocyte located) is present in glycolytic tissues, favouring the formation of lactate from pyruvate (Bittar *et al.* 1996). (3) Glucose is transported across cellular membranes by specific glucose transporters (GLUTs), which are members of the SLC2 family; GLUT1, GLUT3 and GLUT4 are the most abundantly expressed in the brain (Duelli and Kuschinsky 2001). The solute carriers (SLCs) are the largest group of transporters encoded by the human genome (Fredriksson *et al.* 2008), ranging from transporters of inorganic ions, metabolites such as organic acids, amino acids, fatty acids, sugars and purines, and regulatory substances such as neurotransmitters. (4) Monocarboxylate transporters (MCTs, reviewed by Pierre and Pellerin 2005), are composed of 14 members of the SLC16 gene family and are proton-linked membrane carriers involved in the transport of monocarboxylates such as lactate and pyruvate, as well as ketone bodies. In the brain, MCT-1 is expressed by endothelial cells of microvessels as well as by astrocytes. MCT-4 expression appears to be specific for astrocytes, whereas the predominant neuronal isoform is MCT-2. (5) Glutamate is the main excitatory amino acid in the brain, and an ordered coupling between glutamatergic neurons and surrounding glia cells is fundamental for excitatory transmission. Different subtypes of high-affinity glutamate transporter systems participate in this coupling; the EAAT1/GLAST and GLT-1 participate in neuronal protection against excitotoxicity *in vivo* (reviewed by López-Bayghen and Ortega 2011). (6) The brain is unique in that it expresses two separate branched-chain aminotransferase (BCAT) isoenzymes: a peripheral form [mitochondrial (BCATm)] and a form unique to cerebral tissue, placenta and ovaries [cytosolic (BCATc)]. In culture, BCATm functions predominantly in astrocytes and BCATc in neuronal cultures. Metabolic studies indicated that BCAAs promote the efflux of glutamine from astrocytes (Hutson *et al.* 1998).

*Directive*

One purpose of models of biological phenomena is to provide a bridge between disciplines and to facilitate the inter-disciplinary research necessary to understand complex phenomena, of which CNS infection by Mtb is a prime example. In this paper, data from an explorative metabolomics investigation on TBM are used to evaluate the relationship between the intricate spatial network of cell–cell interaction and other characteristics of the CNS with empirical metabolomics data. For this purpose we accepted the physiological role of lactate in homeostasis and in stress as a key player in the paradigms on bioenergetics. Where the conceptual and experimental models agree and disagree will reveal issues for further research. We present the model of the AMLS hypothesis in the hope that it will stimulate directed analytical and systems research into its details, paving the way for improved insights and potential applications in understanding and management of TBM.

|  |  |  |  | **Clinical symptoms (duration in days prior to admission)** | | | |
| --- | --- | --- | --- | --- | --- | --- | --- |
| **Ref** | **TBM stage on admission** | **Chest X-ray evidence of PTB** | **Other evidence of TB outside CNS** | **Fever** | **Vomiting** | **Seizures** | **Poor feeding** |
| 138 | **1** | yes | no | 3 | 3 |  | 3 |
| 140 | **1** | not done | no |  |  |  |  |
| 193 | **1** | no | Positive gastric washing | 1 | 1 |  | 2 |
| 146 | **2a** | yes | not done | 2 | 4 | 2 | 10 |
| 148 | **2a** | no | negative | 14 |  |  | 14 |
| 161 | **2a** | yes | negative | 6 |  |  |  |
| 162 | **2a** | not done | negative | 10 | 1 | 1 | 10 |
| 191 | **2a** | yes | no |  | 14 |  | 7 |
| 229 | **2a** | yes | Positive gastric washing | 8 |  | 1 |  |
| 139 | **2b** | no | no | 1 |  | 1 | 1 |
| 208 | **2b** | no | no | 1 | 1 |  | 5 |
| 216 | **2b** | yes | Positive gastric washing | 3 | 2 |  |  |
| 227 | **2b** | yes | no | 12 | 1 | 1 | 14 |
| 228 | **2b** | no | no | 1 |  | 1 |  |
| 144 | **3** | no | Positive gastric washing | 7 |  |  |  |
| 195 | **3** | no | no | 7 | 2 |  | 7 |
| 211 | **3** | yes | Pos sputum | 3 | 1 | 5 | 3 |
| 221 | **3** | no | no |  | 1 |  |  |
| 226 | **3** | no | no | 21 | 21 |  |  |
| 209 | **3** | no | Trachea aspirate Mtb culture positive | 14 |  |  | 14 |
| 239 | **3** | no | Mandible pus swab Mtb culture positive | 7 | 7 |  |  |

**Table S1**: General clinical symptoms of TBM cases in duration (days) prior to admission to hospital, evidence of TB outside the CNS and stage of TBM.

|  | **Ref.** | **Erythrocytes (cells/µL)** | **Leucocytes (cells/µL)** | **PMN's (cells/µL)** | **Lymphocytes**  **(cells/µL)** | **Protein (g/L)** | **Glucose (mmol/L)** |
| --- | --- | --- | --- | --- | --- | --- | --- |
| **TBM** | 138 | 1320 | 344 | 96 | 238 | 1.49 | 1.2 |
|  | 139 | 39 |  | 7 | 17 | 1.07 | 2.1 |
|  | 140 | 55 | 133 | 28 | 105 | 3 | 2.2 |
|  | 144 | 1 | 40 | 0 | 40 | 0.76 | 2.8 |
|  | 146 | 14 | 440 | 30 | 410 | 2.1 | 0.6 |
|  | 148 | 28 | 199 | 6 | 193 | 2 | 0.5 |
|  | 161 | 0 | 29 | 0 | 29 | 1.24 | 0.5 |
|  | 162 | 4 | 172 | 15 | 157 | 0.72 | 1.4 |
|  | 191 | 0 | 27 | 0 | 27 | 3 | 0.2 |
|  | 193 |  | 92 | 12 | 80 | 0.94 | 1.6 |
|  | 195 | 1875 | 349 | 2 | 347 | 1.09 | 2 |
|  | 208 | 0 | 11 | 0 | 11 | 0.96 | 1.5 |
|  | 209 | 155 | 30 | 0 | 30 |  |  |
|  | 211 | 0 | 0 | 0 | 0 | 1.2 | 3.9 |
|  | 216 | 83 | 270 | 33 | 237 | 0.94 | 1.1 |
|  | 221 | 5 | 32 | 6 | 26 | 3 | 2.5 |
|  | 226 | 220 | 7 | 2 | 5 | 0.21 |  |
|  | 227 | 462 | 9 | 0 | 9 |  |  |
|  | 228 | 0 | 0 | 0 | 0 | 0.62 | 5.1 |
|  | 229 | 100 | 0 | 0 | 21 | 0.87 | 2.7 |
|  | 239 | 0 | 1 | 1 | 0 | 1.57 | 3 |
| **SA_Control** | C143 | 0 | 0 | 0 | 0 | 0.15 | 3.8 |
|  | C149 | 0 | 0 | 0 | 0 | 0.24 | 4.2 |
|  | C151 | 0 | 0 | 0 | 0 | 0.14 | 3.7 |
|  | C159 | 1 | 2 | 0 | 2 | 0.14 | 4.0 |
|  | C171 | 0 | 2 | 0 | 2 | 0.3 | 3.7 |
|  | C173 | 0 | 0 | 0 | 0 | 0.11 | 3.9 |
|  | C184 | 0 | 0 | 0 | 0 | 0.32 | 4.6 |
|  | C190 | 0 | 0 | 0 | 0 | 0.11 | 3.9 |
|  | C192 | 0 | 0 | 0 | 0 | 0.11 | 3.3 |
|  | C199 | 0 | 0 | 0 | 0 |  | 4.1 |
|  | C203 | 0 | 2 | 0 | 2 | 0.25 | 4.0 |
|  | C204 | 0 | 0 | 0 | 0 | 0.09 | 4.6 |
|  | C206 | 0 | 0 | 0 | 0 | 0.27 | 5.2 |
|  | C212 | 0 | 1 | 0 | 1 | 0.18 | 3.1 |
|  | C220 | 0 | 0 | 0 | 0 | 0.18 | 3.7 |
|  | C222 | 0 | 0 | 0 | 0 | 0.25 | 3.6 |
|  | C232 | 1 | 1 | 0 | 1 | 0.34 | 4.0 |
|  | C236 | 0 | 34 | 0 | 34 | 0.32 | 4.6 |
|  | C240 | 0 | 0 | 0 | 0 | 0.23 | 4.3 |

**Table S2**: Clinical chemistry of CSF describing elevated immuno-cells, decreased glucose and elevated proteins in TBM cases compared to SA_Control cases.

|  | **Ref.** | **Total medication** | **TB-specific** | **Antibiotic** | **Analgesic** | **Anti-inflammatory** | **Anti-seizure** | **Sedative** | **Other** |
| --- | --- | --- | --- | --- | --- | --- | --- | --- | --- |
| **TBM** | **138** | 0 |  |  |  |  |  |  |  |
|  | **140** | 0 |  |  |  |  |  |  |  |
|  | **144** | 0 |  |  |  |  |  |  |  |
|  | **195** | 0 |  |  |  |  |  |  |  |
|  | **229** | 0 |  |  |  |  |  |  |  |
|  | **146** | 1 |  | 1 |  |  |  |  |  |
|  | **161** | 5 | 4 |  |  | 1 |  |  |  |
|  | **191** | 5 | 4 |  |  | 1 |  |  |  |
|  | **211** | 6 | 4 | 1 |  | 1 |  |  |  |
|  | **227** | 6 | 3 | 1 |  | 1 |  | 1 |  |
|  | **228** | 6 | 4 | 2 |  |  |  |  |  |
|  | **148** | 7 | 4 | 1 | 1 | 1 |  |  |  |
|  | **209** | 7 | 4 | 1 | 1 | 1 |  |  |  |
|  | **162** | 8 | 4 | 2 |  | 1 |  |  | 1 |
|  | **221** | 8 | 4 | 1 |  |  |  |  | 3 |
|  | **193** | 9 | 4 | 2 | 1 | 1 |  | 1 | 1 |
|  | **139** | 10 | 4 | 2 |  | 1 | 1 |  | 2 |
|  | **208** | 10 | 4 | 2 | 1 | 2 |  |  | 1 |
|  | **216** | 10 | 4 | 1 | 1 | 1 |  |  | 3 |
|  | **226** | 11 | 4 | 1 | 1 | 2 |  | 1 | 2 |
|  | **239** | 14 | 3 | 4 | 1 | 3 |  |  | 2 |
| **SA_Control** | **c149** | 0 |  |  |  |  |  |  |  |
|  | **c143** | 2 | 1 | 1 |  |  |  |  |  |
|  | **c151** | 2 |  |  | 1 |  |  |  | 1 |
|  | **c203** | 3 |  | 2 |  |  |  |  | 1 |
|  | **c159** | 4 |  | 2 | 1 |  |  | 1 |  |
|  | **c190** | 4 |  | 1 |  |  |  | 1 | 2 |
|  | **c212** | 4 |  | 2 | 1 |  |  |  | 1 |
|  | **c236** | 4 |  | 1 | 1 |  | 1 |  | 1 |
|  | **c240** | 4 |  | 2 | 1 |  |  | 1 |  |
|  | **c173** | 5 |  | 3 | 1 |  |  |  | 1 |
|  | **c199** | 6 |  | 2 | 1 |  |  | 2 | 1 |
|  | **c204** | 6 | 3 | 1 | 1 | 1 |  |  |  |
|  | **c184** | 7 | 4 | 1 | 1 | 1 |  |  |  |
|  | **c171** | 9 | 4 | 3 |  | 1 |  |  | 1 |
|  | **c192** | 9 | 2 | 1 |  |  |  |  | 6 |
|  | **c206** | 9 |  | 2 | 1 | 1 |  | 1 | 4 |
|  | **c220** | 10 | 3 | 3 |  | 1 | 1 |  | 2 |
|  | **c222** | 10 | 4 | 2 | 1 |  |  | 1 | 2 |
|  | **c232** | 10 |  | 5 | 1 | 1 |  |  | 3 |

**Table S3**: Summary of total, and type of, medication administered to the TBM and SA_Control cases, analysed in this study, prior to admission to hospital. Medications classified as: (i) **TB-specific** (Pyrazinamide, Rifampicin, Ethionamide, Ethambutol, Isoniazid, Rimcure); (ii) **Antibiotic** (Cephtriaxone, Acyclovir, Amoxicillin, Vancomycin, Ciprofloxacin, Cloxacillin, Coamoxiclav, Ampicillin, Metronidazole, Penicillin, Flucloxacillin); (iii) **Analgesic** (Paracetamol); (iv) **Anti-inflammatory** (Prednisone, Ibuprofen, Dexamethasone, Solu-Medrol); (v) **Anti-seizure** (Phenobarbital); (vi) **Sedative** (Midazolam, Ketamine, chloral hydrate, Diazepam); and (vii) **Other** (Lasix (diuretic), mannitol (diuretic), Sorbitol (laxative), Allergex, Diamox (carbonic anhydrase inhibitor), Albendazole (Anti-Worm), Valeron (muscle relaxant), Haloperidol (anti-psychotic), Nystatin (anti-fungal), Illidian Drops (decongestant), multivitamin and other supplements (pyridoxine, zinc, potassium chloride, vitamin A, glycerine, lactulose)).

| **Variables (SA_Controls vs TBM)** | **Power** |  | **Variables (NL_Controls vs TBM)** | **Power** |
| --- | --- | --- | --- | --- |
| ^13^C Lactate (1.27) | 0.931390 |  | lactate (1.41) | 0.918325 |
| glucose (3.86) | 0.878166 |  | glucose (3.24) | 0.886187 |
| lactate (4.36) | 0.872698 |  | glucose (3.86) | 0.885595 |
| glucose (3.71) | 0.867672 |  | glucose (3.48) | 0.874681 |
| lactate (1.41) | 0.866338 |  | lactate (4.36) | 0.872905 |
| glucose (3.24) | 0.861590 |  | glucose (4.63) | 0.871041 |
| ^13^C Lactate (1.53) | 0.859754 |  | glucose (3.38) | 0.868400 |
| ^13^C Lactate (1.283) | 0.857551 |  | glucose (3.71) | 0.867712 |
| glucose (3.48) | 0.849267 |  | glucose (3.21) | 0.861035 |
| glucose (3.38) | 0.847711 |  | glucose (5.22) | 0.847384 |
| glucose (5.22) | 0.844244 |  | glucose/glycerol (3.43) | 0.800225 |
| glucose (3.21) | 0.838305 |  | Pyrazinamide (8.81) | 0.763011 |
| glucose (4.63) | 0.824405 |  | Pyrazinamide (8.75) | 0.731714 |
| glucose/glycerol (3.43) | 0.822220 |  | Pyrazinamide (9.18) | 0.713093 |
| alanine (1.51) | 0.781326 |  | alanine (1.51) | 0.687848 |
| acetate (2.08) | 0.759398 |  | 3OHisovalerate/threonine (1.33) | 0.672409 |
| valine 1.04) | 0.758630 |  | ^13^C Lactate (1.283) | 0.635886 |
| lysine (1.73) | 0.748802 |  | ^13^C Lactate (1.27) | 0.621801 |
| 1.0255 | 0.723804 |  | creatinine (3.13) | 0.618343 |
| creatine (3.05) | 0.685460 |  | valine/isoleucine (1.01) | 0.613439 |
| choline (3.19) | 0.678086 |  | 1.93715 | 0.600289 |
| 3.03299999 | 0.668447 |  | 8.70874977 | 0.600242 |
| 1.93715 | 0.668090 |  | phenylalanine (7.42) | 0.578138 |
| myo-inositol (4.05) | 0.662570 |  | 8.77799988 | 0.573148 |
| 7.96000004 | 0.662132 |  | myo-inositol (3.285) | 0.563730 |
| 3.56700003 | 0.658366 |  | ^13^C Lactate (1.53) | 0.548962 |
| 1.29099995 | 0.652387 |  | 3.14499998 | 0.546544 |
| Pyrazinamide (9.18) | 0.631420 |  | valine 1.04) | 0.546472 |
| 1.27685004 | 0.628092 |  | myo-inositol (4.05) | 0.535355 |
| valine/isoleucine (1.01) | 0.624230 |  | tyrosine (7.19) | 0.532997 |
| 7.99500012 | 0.620387 |  | 9.19474983 | 0.526925 |
| 8.66499996 | 0.613486 |  | 3.59799993 | 0.524536 |
| Pyrazinamide (8.81) | 0.612643 |  | Isoniazid (8.94) | 0.522664 |
| formate (8.25) | 0.608682 |  | 7.96000004 | 0.521607 |
| Pyrazinamide (8.75) | 0.597625 |  | 8.09674978 | 0.512562 |
| creatine (4.10) | 0.586217 |  |  |  |
| 2.00975001 | 0.582802 |  |  |  |
| 7.77749991 | 0.579755 |  |  |  |
| 3OHbutyrate (2.53) | 0.574481 |  |  |  |
| myo-inositol (3.285) | 0.573663 |  |  |  |
| creatinine (3.13) | 0.564981 |  |  |  |
| tyrosine (7.19) | 0.532237 |  |  |  |
| Acyclovir (5.55) | 0.527150 |  |  |  |
| 8.09674978 | 0.505257 |  |  |  |
| 3.14499998 | 0.505217 |  |  |  |

**Table S4**: PCA power values of SA_Controls vs TBM and NL_Controls vs TBM cases for all 109 variables, with a cutoff value of 0.5, illustrating quantitatively that lactate and glucose were the two dominant discriminating metabolites.

D

| SA_Controls vs TBM | PLS-DA | Fold change | t-test | Validation |  | NL_Controls vs TBM | PLS-DA | Fold Change | t-test | Validation |
| --- | --- | --- | --- | --- | --- | --- | --- | --- | --- | --- |
|  | VIP | d-value | p-value | p-values |  |  | VIP | d-value | p-value | p-value |
| lactate (1.41) | 1.631 | 3.172 | <0.001 | <0.001 |  | lactate (1.41) | 1.779 | 2.831 | <0.001 | <0.001 |
| lactate (4.36) | 1.630 | 3.032 | <0.001 | <0.001 |  | lactate (4.36) | 1.755 | 2.584 | <0.001 | <0.001 |
| ^13^C lactate (1.27) | 1.538 | 2.931 | <0.001 | <0.001 |  | glucose (4.63) | 1.663 | 0.303 | <0.001 | <0.001 |
| ^13^C lactate (1.53) | 1.471 | 2.536 | <0.001 | <0.001 |  | glucose (3.24) | 1.584 | 0.493 | <0.001 | <0.001 |
| ^13^C lactate (1.283) | 1.470 | 2.817 | <0.001 | <0.001 |  | glucose (3.86) | 1.574 | 0.611 | <0.001 | <0.001 |
| glucose (3.86) | 1.425 | 0.571 | <0.001 | <0.001 |  | glucose (3.71) | 1.573 | 0.549 | <0.001 | <0.001 |
| glucose/glycerol (3.43) | 1.413 | 0.582 | <0.001 | <0.001 |  | glucose (3.48) | 1.557 | 0.500 | <0.001 | <0.001 |
| glucose (3.71) | 1.413 | 0.526 | <0.001 | <0.001 |  | glucose (3.21) | 1.539 | 0.503 | <0.001 | <0.001 |
| glucose (3.24) | 1.407 | 0.467 | <0.001 | <0.001 |  | glucose (3.38) | 1.531 | 0.493 | <0.001 | <0.001 |
| glucose (3.48) | 1.396 | 0.464 | <0.001 | <0.001 |  | glucose (5.22) | 1.514 | 0.482 | <0.001 | <0.001 |
| choline (3.19) | 1.392 | 4.736 | <0.001 | <0.001 |  | ^13^C lactate (1.283) | 1.479 | 3.055 | <0.001 | <0.001 |
| alanine (1.51) | 1.388 | 3.351 | <0.001 | <0.001 |  | glucose/glycerol (3.43) | 1.476 | 0.658 | <0.001 | <0.001 |
| glucose (5.22) | 1.385 | 0.440 | <0.001 | <0.001 |  | alanine (1.51) | 1.437 | 3.448 | <0.001 | <0.001 |
| glucose (3.38) | 1.374 | 0.455 | <0.001 | <0.001 |  | valine (1.04) | 1.392 | 3.454 | <0.001 | <0.001 |
| glucose (3.21) | 1.370 | 0.482 | <0.001 | <0.001 |  | ^13^C lactate (1.27) | 1.378 | 2.881 | <0.001 | <0.001 |
| glucose (4.63) | 1.331 | 0.440 | <0.001 | <0.001 |  | creatinine (3.13) | 1.326 | 0.570 | <0.001 | <0.001 |
| lysine (1.73) | 1.295 | 2.053 | <0.001 | <0.001 |  | DMSO_2_ (3.14) | 1.255 | 0.322 | <0.001 | <0.001 |
| valine/isoleucine (1.01) | 1.242 | 2.940 | <0.001 | <0.001 |  | ^13^C lactate (1.53) | 1.207 | 2.451 | <0.001 | <0.001 |
| valine (1.04) | 1.173 | 2.867 | <0.001 | <0.001 |  | lysine (1.73) | 1.119 | 1.868 | <0.001 | <0.001 |
| glucose/glycerol (3.54) | 1.151 | 0.704 | <0.001 | <0.001 |  | valine/isoleucine (1.01) | 1.107 | 2.381 | <0.001 | 0.0001 |
| creatine (3.05) | 1.113 | 1.502 | <0.001 | <0.001 |  | creatinine (4.29) | 1.092 | 0.600 | <0.001 | <0.001 |
| isoleucine/leucine (0.95) | 1.075 | 1.784 | <0.001 | <0.001 |  | myoinositol (3.285) | 1.052 | 0.611 | <0.001 | 0.0007 |
| pyruvate (1.56) | 1.023 | 1.915 | <0.001 | 0.0003 |  | tyrosine/medication (6.90) | 1.009 | 1.678 | <0.001 | 0.0001 |
| pyruvate (2.37) | 1.001 | 1.777 | <0.001 | 0.0012 |  | ethanol (1.18) | 1.005 | 4.488 | <0.001 | 0.0009 |
| formate (8.25) | 0.955 | 1.948 | <0.001 | <0.001 |  | myo-inositol (4.05) | 0.911 | 0.603 | 0.001 | 0.0075 |
| creatinine (3.13) | 0.945 | 0.707 | <0.001 | <0.001 |  | tyrosine (7.19) | 0.901 | 1.537 | 0.001 | 0.0001 |
| threonine (1.34) | 0.933 | 1.707 | <0.001 | <0.001 |  | phenylalanine (7.39) | 0.819 | 1.891 | 0.002 | 0.0008 |
| tyrosine (7.19) | 0.919 | 1.805 | 0.001 | <0.001 |  | isoleucine/leucine (0.95) | 0.788 | 1.404 | 0.004 | 0.0007 |
| carnitine (3.22) | 0.874 | 1.379 | 0.001 | 0.0003 |  | phenylalanine (7.42) | 0.776 | 1.465 | 0.004 | 0.0051 |
| 2-oxoglutarate (2.68) | 0.820 | 2.190 | 0.002 | 0.0015 |  | glucose/glycerol (3.54) | 0.745 | 0.826 | 0.006 | 0.0048 |
| citrate (3.00) | 0.811 | 1.234 | 0.003 | 0.0001 |  | choline (3.19) | 0.710 | 1.505 | 0.010 | 0.0066 |
| phenylalanine (7.42) | 0.784 | 1.739 | 0.004 | 0.0004 |  | creatine (3.05) | 0.654 | 1.225 | 0.018 | 0.0100 |
| betaine/myoinositol (3.27) | 0.769 | 1.309 | 0.005 | <0.001 |  | acetoacetate (2.30) | 0.615 | 0.915 | 0.026 | 0.0131 |
| acetate (2.08) | 0.755 | 0.744 | 0.006 | 0.0435 |  | pyruvate (1.56) | 0.596 | 1.217 | 0.031 | 0.0492 |
| myo-inositol (3.285) | 0.752 | 0.793 | 0.006 | 0.0021 |  | pyruvate (2.37) | 0.579 | 1.209 | 0.037 | 0.0650 |
| phenylalanine (7.39) | 0.712 | 1.775 | 0.010 | 0.0084 |  | creatine (4.10) | 0.354 | 0.878 | 0.209 | 0.4470 |
| succinate (2.66) | 0.635 | 1.830 | 0.022 | 0.0014 |  | glutamine (2.47) | 0.353 | 0.901 | 0.209 | 0.1119 |
| DMSO_2_ (3.14) | 0.620 | 0.637 | 0.026 | 0.0143 |  | citrate (2.84) | 0.353 | 0.852 | 0.210 | 0.3389 |
| tyrosine/medication (6.90) | 0.601 | 1.327 | 0.031 | 0.0532 |  | 3-OHbutyrate (2.53) | 0.348 | 0.973 | 0.216 | 0.2698 |
| myo-inositol (4.05) | 0.559 | 0.819 | 0.046 | 0.0726 |  | mannose (5.17) | 0.334 | 0.219 | 0.236 | 0.0886 |
| 3-OHisovalerate/threonine (1.33) | 0.511 | 1.321 | 0.070 | 0.0055 |  | citrate (2.97) | 0.321 | 1.063 | 0.255 | 0.2415 |
| citrate (2.97) | 0.502 | 1.149 | 0.075 | 0.0944 |  | citrate (3.00) | 0.313 | 1.059 | 0.267 | 0.1507 |
| acetoacetate (2.30) | 0.456 | 1.227 | 0.107 | 0.0622 |  | carnitine (3.22) | 0.267 | 1.101 | 0.345 | 0.3390 |
| glutamine/medication (2.16) | 0.436 | 1.103 | 0.124 | 0.2702 |  | acetate (2.08) | 0.253 | 0.898 | 0.370 | 0.5321 |
| creatinine (4.29) | 0.388 | 0.971 | 0.172 | 0.0086 |  | citrate (2.81) | 0.248 | 1.029 | 0.380 | 0.3189 |
| ethanol (1.18) | 0.383 | 1.522 | 0.178 | 0.1720 |  | formate (8.25) | 0.221 | 1.138 | 0.435 | 0.2261 |
| 3-OHbutyrate (2.53) | 0.352 | 1.080 | 0.216 | 0.1007 |  | glutamine/medication (2.16) | 0.217 | 1.042 | 0.443 | 0.8327 |
| acetone (2.22) | 0.193 | 0.569 | 0.501 | 0.4334 |  | succinate (2.66) | 0.126 | 0.967 | 0.656 | 0.2014 |
| glutamine (2.47) | 0.156 | 1.059 | 0.587 | 0.9259 |  | 3-OHbutyrate (1.23) | 0.113 | 0.734 | 0.691 | 0.4416 |
| 3-OHbutyrate (1.23) | 0.130 | 0.935 | 0.651 | 0.7012 |  | 3-OHisovalerate/threonine (1.33) | 0.087 | 0.937 | 0.759 | 0.9328 |
| mannose (5.17) | 0.126 | 0.776 | 0.662 | 0.5105 |  | 2-oxoglutarate (2.68) | 0.086 | 0.938 | 0.762 | 0.8473 |
| ethanol (3.64) | 0.079 | 1.128 | 0.783 | 0.5900 |  | acetone (2.22) | 0.068 | 0.574 | 0.810 | 0.8454 |
| citrate (2.81) | 0.054 | 0.996 | 0.851 | 0.9574 |  | betaine/myoinositol (3.27) | 0.035 | 0.966 | 0.902 | 0.8699 |
| creatine (4.10) | 0.045 | 0.982 | 0.875 | 0.4468 |  | threonine (1.34) | 0.021 | 1.015 | 0.940 | 0.8168 |
| citrate (2.84) | 0.008 | 1.009 | 0.979 | 0.7428 |  | ethanol (3.64) | 0.016 | 1.082 | 0.954 | 0.9525 |

**Table S5**: Quantitative data for all identified variables, indicating the most important metabolites in discriminating between TBM and non-TBM for both SA_Controls vs TBM and NL_Controls vs TBM cases; which includes: PLS-DA VIP values, fold change d-values and p-values for the t-test and from the cross-validation of the PLS model. [the chemical shift, in ppm, of each identified metabolite given in brackets]


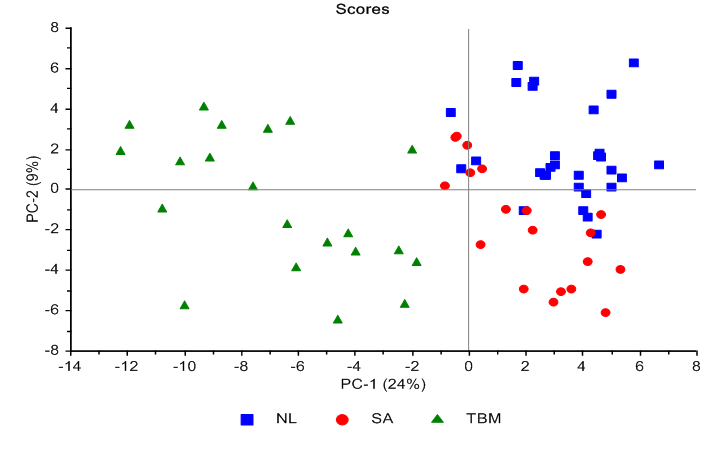


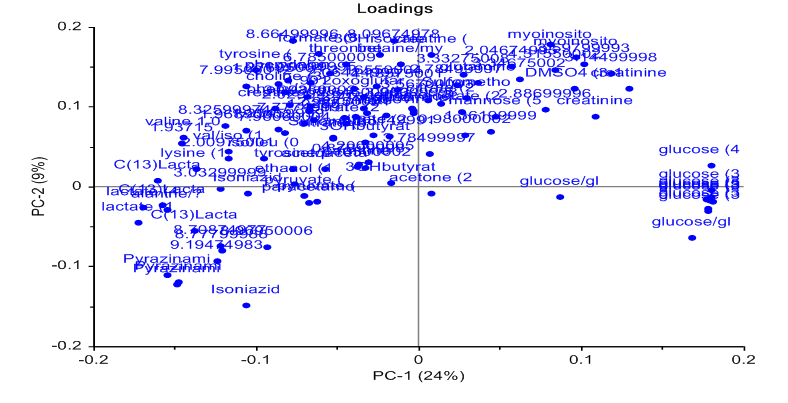


**Figure S1**: PCA scores plot (above) showing natural separation between TBM and non-TBM cases, with overlapping between control groups, and associated loadings plot (below) for all 109 variables.


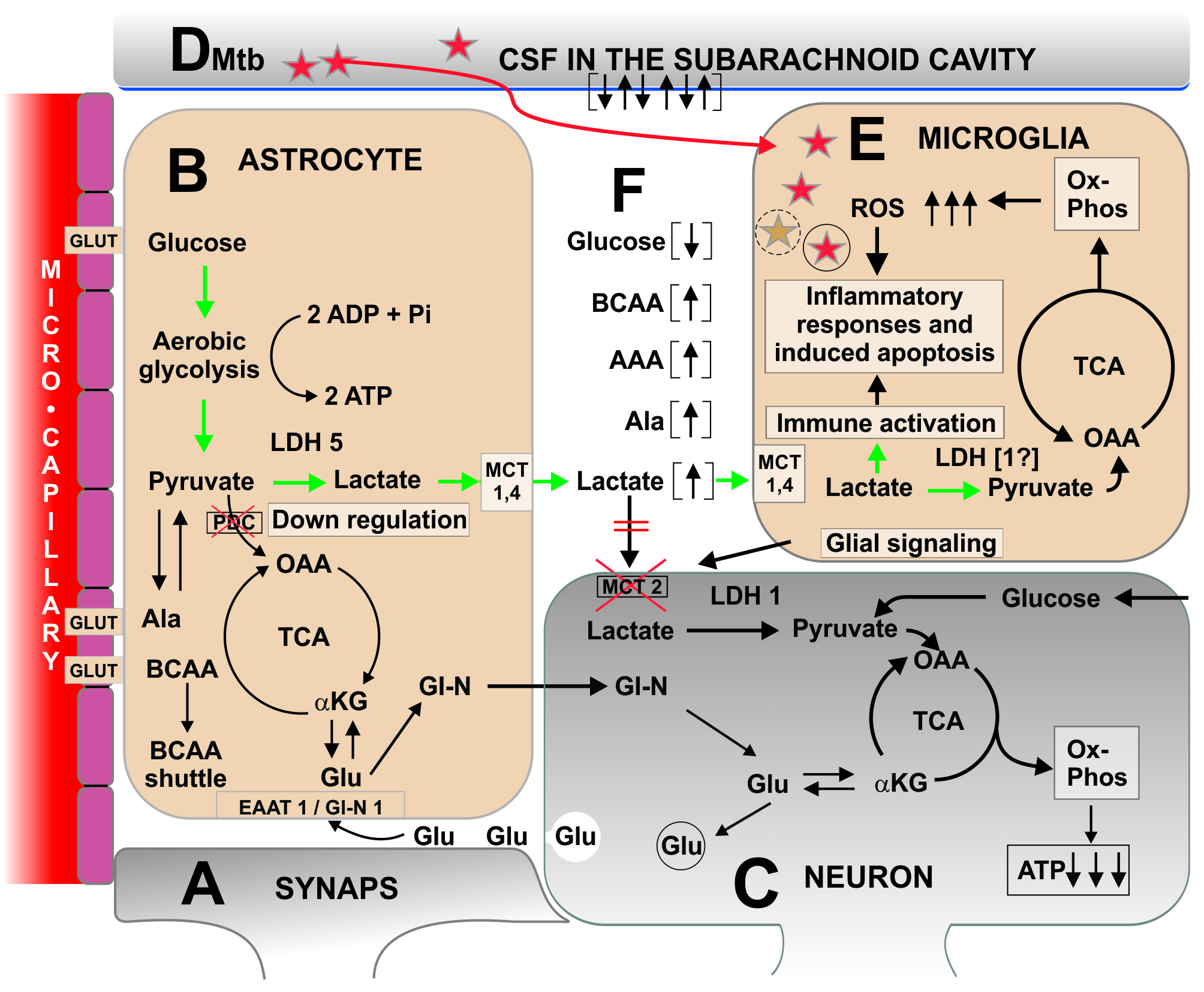


**Figure S2:** Conceptual model of the hypothetical “astrocyte–microglia lactate shuttle”.

Legend:

**1.** **Energy profile during homeostasis and neuron activation.** Lactate shuttles provide models describing the movement of intra- and intercellular lactate between diverse cells in several tissues under both anaerobic and aerobic conditions. An “astrocyte–neuron lactate shuttle” in the brain is the model for energy cooperation between astrocytes and neurons during high energy demand in the neurons. During homeostasis and neuron activation, an important energy load of the neurons is required for the operation of glutamatergic synapses [**A**], which is tightly regulated by intercellular links and dynamic interactions between astrocytes and neurons. The glutamate-induced glucose uptake in the astrocytes by endothelial GLUT1 transporters [**B**], followed by glycolysis, functions as the cellular mechanism coupling the astrocytes’ energy production to neuronal energy-dependent activity [**B** and **C**]**.** A putative branched-chain amino acid (BCAA) shuttle, involving isoforms of branched-chain aminotransferases where BCAAs provide the amino group for glutamate synthesis from α-ketoglutarate, promote glutamine transfer to neurons. In their turn, the neurons re-aminate the amino acids for another cycle of the shuttle. Decreased aerobic oxidation (TCA and oxidative phosphorylation) in the astrocytes during neuron activation results from down regulation of the PDC through phosphorylation of its PDHα subunit. The LDH of astrocytes is primarily of the type-5 isoform (LDH-5), which catalyzes pyruvate hydrogenation to lactate, the final catabolic end product of astrocytes’ aerobic glycolysis. Neurons primarily express LDH-1, which catalyzes lactate to pyruvate. Furthermore, astrocytes, as well as neurons, express MCT proteins participating in the lactate shuttle. MCT-4 in astrocytes is a low affinity transporter for lactate (high K_m_: facilitates export of lactate produced by glycolysis in the astrocytes). Neurons express MCT-2, a high affinity transporter for lactate (low K_m_: facilitates the uptake of lactate produced by the astrocytes). Consistent with their higher energy requirements, activated neurons sustain a high rate of oxidative metabolism, emanating from lactate, which enters the neuronal TCA cycle via pyruvate, neuronal oxidative phosphorylation and ATP production.

**2. Energy profile during TBM neuroinflammation.** In TBM, the causative Mtb apparently enters the subarachnoid space [**D**], followed by its distinctive capacity to enter and replicate within the microglial cell [**E**], which, however, also provides the first line of defence against invading pathogens. Brain cells are surrounded by interstitial fluid [**F**], which is contiguous with CSF in the subarachnoid space [**D**]. The profile of energy-associated metabolites in CSF from TBM patients differs significantly from that of these metabolites associated with the astrocyte–neuron–lactate shuttle, leading to the present hypothesis of an “astrocyte–microglia lactate shuttle” as operating in neuroinflammation, triggered by Mtb infection. It is proposed that an analogue of the reverse Warburg process underpins this shuttle. Bidirectional communication networks between glial cells and neurons may produce signalling substances directed to downregulation of neuronal MCT-2 and of the BCAA shuttle for high lactate and keto acids provision to neurons. Stimulated by proinflammatory signals, including from lactate, the microglia soon undergo an array of transformations, resulting in an oxidative burst that produces superoxide from an orchestrated mechanism involving nicotinamide adenine dinucleotide phosphate (NADPH) oxidase, leading to the formation of reactive oxygen species (ROS) required for bactericidal activity and eradication of pathogens. However, activation of microglia may likewise compromise their viability, and some of this vulnerability possibly comes from the oxidative stress of the oxidative burst and a perturbed ROS balance. Activated microglia finally secrete a diverse array of proinflammatory molecules, suggesting that oxidative stress emanating from activated microglia affects the astrocytes, causes loss of neuron function and eventually precipitates the severe clinical symptoms of TBM.


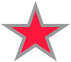
 Mtb;
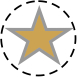
 destroyed Mtb;
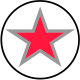
 protected Mtb;
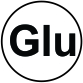
 neuronal glutamate vesicle; GLUT, microvascular transporters for glucose, organic acids, fatty acids, amino acids and ketones; PDC, pyruvate dehydrogenase complex; MCT, monocarboxylate transporter; EAAT 1/Gl-N 1, transporters for glutamate; Ala, alanine; BCAA, branched-chain amino acids; AAA, aromatic amino acids; Glu, glutamate; Gl-N, glutamine; OAA, oxalic acetic acid; αKG, α-ketoglutaric acid; Ox-Phos, oxidative phosphorylation; ROS, reactive oxygen species; CSF, cerebrospinal fluid.


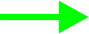
Direction of the trafficking/energy flow in the “astrocyte–microglia–lactate shuttle”

**References**

Abbott, N.J., Rönnbäck, L., Hansson, E. (2006). Astrocyte–endothelial interactions at the blood–brain barrier. *Nature Reviews – Neuroscience*, *7*, 41–53.

Allaman, I., Bélanger, M., Magistretti, P.J. (2011). Astrocyte–neuron metabolic relationships: For better or for worse. *Trends in Neurosciences*, *11*, 76–87.

Bittar, P.G., Charnay, Y., Pellerin, L., Bouras, C., Magistretti, P.J. (1996). Selective distribution of lactate dehydrogenase isoenzymes in neurons and astrocytes of human brain. *Journal of Cerebral Blood Flow and Metabolism*, *16*, 1079–1089.

Chen, Y. and Swanson, R.A. (2003). Astrocytes and brain injury. *Journal of Cerebral Blood Flow & Metabolism*, *23*, 137–149.

Craig, A., Cloarec, O., Holmes, E., Nicholson, J.K., Lindon, J.C. (2006). Scaling and normalization effects in NMR spectroscopic metabonomic data sets. *Analytical Chemistry*, *78(7),* 2262–2267.

Duelli, R. and Kuschinsky, W. (2001). Brain glucose transporters: Relationship to local energy demand. *Physiology*, *16*, 71–76.

Ellinger, J.J., Chylla, R.A., Ulrich, E.L., Markley, J.L. (2013). Databases and software for NMR-based metabolomics. *Current Metabolomics*, *1*, 28–40.

Engelke, U.F.H., Tangerman, A., Willemsen, M.A.A.P., Moskau, D., Loss, S., Mudd, S.H., Wevers, R.A. (2005). Dimethyl sulfone in human cerebrospinal fluid and blood plasma confirmed by one-dimensional ^1^H and two-dimensional ^1^H-^13^C NMR. *NMR in Biomedicine*, *18*, 331–336.

Engelke, U.F.H., Kremer, B., Kluijtmans, L.A.J., van der Graaf, M., Morava, E., Loupatty, F.J., Wanders, R.J.A., Moskau, D., Loss, S., van den Bergh, E., Wevers, R.A. (2006). NMR spectroscopic studies on the late onset form of 3-methylglutaconic aciduria type I and other defects in leucine metabolism. *NMR in Biomedicine*, *19*, 271–278.

Fredriksson, R., Nordström, K.J.V., Stephansson, O., Hägglund, M.G.A., Schiöth, H.B. (2008). The solute carrier (SLC) complement of the human genome: Phylogenetic classification reveals four major families. *FEBS Letters*, *582*, 3811–3816.

Goel, G., Chou, I.C., Voit, E.O. (2006). Biological systems modeling and analysis: A biomolecular technique of the twenty-first century. *Journal of Biomolecular Techniques, JBT*, *17*, 252–269.

Halim, N.D., McFate, T., Mohyeldin, A., Okagaki, P., Korotchlina, L.G., Patel, M.S., Jeoung, N.H., Harris, R.A., Schell, M.J., Verma, A. (2010). Phosphorylation status of pyruvate dehydrogenase distinguishes metabolic phenotypes of cultured rat brain astrocytes and neurons. *Glia*, *58,* 1168–1176.

Hutson, S.M., Berkich, D., Drown, P., Xu, B., Aschner, M., LaNoue, K.F. (1998). Role of branched-chain aminotransferase isoenzymes and gabapentin in neurotransmitter metabolism. *Journal of Neurochemistry*, *71*, 863–874.

Lin, C.Y., Wu, H., Tjeerdema, R.S., Viant, M.R. (2007). Evaluation of metabolite extraction strategies from tissue samples using NMR metabolomics. *Metabolomics*, *3*, 55–67.

López-Bayghen, E. and Ortega, A. (2011). Glial glutamate transporters: New actors in brain signaling. *IUBMB Life*, *63*, 816–823.

Maddula, S. and Baumbach, J.I. (2011). Heterogeneity in tumor cell energetic metabolome at different cell cycle phases of human colon cancer cell lines. *Metabolomics, 7*, 509–523.

Merrill, J.E. and Jonakait, G.M. (1995). Interactions of the nervous and immune systems in development, normal brain homeostasis, and disease. *FASEB Journal, 9*, 611–618.

Nareika, A., He, L., Game, B.A., Slate, E.H., Sanders, J.J., London, S.D., Lopes-Virella, M.F., Huang, Y. (2005). Sodium lactate increases LPS-stimulated MMP and cytokine expression in U937 histiocytes by enhancing AP-1 and NF-kappaB transcriptional activities. *American Journal of Physiology, Endocrinology and Metabolism, 289*, E534–E542.

Pan, Z., Gu, H., Talaty, N., Chen, H., Shanaiah, N., Hainline, B.E., Cooks, R.G., Raftery, D. (2007). Principal component analysis of urine metabolites detected by NMR and DESI-MS in patients with inborn errors of metabolism. *Analytical and Bioanalytical Chemistry*, *387*, 539–549.

Pavlides, S., Whitaker-Menezes, D., Castello-Cros, R., Flomenberg, N., Witkiewicz, A.K., Frank, P.G., Casimiro, M.C., Wang, C., Fortina, P., Addya, S., Pestell, R.G., Martinez-Outschoorn, U.E., Sotgia, F., Lisanti, M.P. (2009). The reverse Warburg effect - aerobic glycolysis in cancer associated fibroblasts and the tumor stroma. *Cell Cycle*, *8*, 3984–4001.

Pavlides, S., Tsirigos, A., Vera, I., Flomenberg, N., Frank, P.G., Casimiro, M.C., Wang, C., Pestell, R.G., Martinez‐Outschoorn, U.E., Howell, A., Sotgia, F., Lisanti, M.P. (2010). Transcriptional evidence for the “Reverse Warburg Effect” in human breast cancer tumor stroma and metastasis: Similarities with oxidative stress, inflammation, Alzheimer’s disease, and “Neuron‐Glia Metabolic Coupling”. *Aging*, *2*, 185–199.

Pears, M.R., Cooper, J.D., Mitchison, H.M., Mortishire-Smith, R.J., Pearce, D.A., Griffin, J.L. (2005). High resolution ^1^H NMR-based metabolomics indicates a neurotransmitter cycling deficit in cerebral tissue from a mouse model of batten disease. *The Journal of Biological Chemistry*, *280(52)*, 42508–42514.

Pellerin, L. and Magistretti, P.J. (1994). Glutamate uptake into astrocytes stimulates aerobic glycolysis: A mechanism coupling neuronal activity to glucose utilization. *Proceedings of the National Academy of Sciences of the USA*, *91*, 10625–10629.

Pellerin, L., Pellegri, G., Bittar, P.G., Charnay, Y., Bouras, C., Martin, J.L., Stella, N., Magistretti, P.J. (1998). Evidence supporting the existence of an activity-dependent astrocyte-neuron lactate shuttle. *Developmental Neuroscience, 20*, 291–299.

Peterson, P.K., Gekker, G., Hu, S., Sheng, W.S., Anderson, W.R., Ulevitch, R.J., Tobias, P.S., Gustafson, K.W., Molitor, T.W., Chao, C.C. (1995). CD14 receptor-mediated uptake of nonopsonized *Mycobacterium tuberculosis* by human microglia. *Infection and Immunity*, *63*, 1598–1602.

Pierre, K. and Pellerin, L. (2005). Monocarboxylate transporters in the central nervous system: Distribution, regulation and function. *Journal of Neurochemistry*, *94*, 1–14.

Powers, R. (2008). NMR metabolomics and drug discovery. *Magnetic Resonance in Chemistry*, *47*, S2–S11.

Purohit, P.V., Rocke, D.M., Viant, M.R., Woodruff, D.L. (2004). Discrimination models using variance-stabilizing transformation of metabolomic NMR data. *OMICS: A Journal of Integrative Biology*, *8(2)*, 118–130.

Sinclair, A.J., Viant, M.R., Ball, A.K., Burdon, M.A., Walker, E.A., Stewart, P.M., Rauz, S., Young, S.P. (2009). NMR-based metabolomic analysis of cerebrospinal fluid and serum in neurological diseases – a diagnostic tool? *NMR in Biomedicine*, *23*, 123–132.

Viant, M.R., Lyeth, B.G., Miller, M.G., Berman, R.F. (2005). An NMR metabolomic investigation of early metabolic disturbances following traumatic brain injury in a mammalian model. *NMR in Biomedicine*, *18*, 507-516.

Waters, N.J., Holmes, E., Williams, A., Waterfield, C.J., Farrant, R.D., Nicholson, J.K. (2001). NMR and pattern recognition studies on the time-related metabolic effects of α-napthylisothiocyanate on liver, urine, and plasma in the rat: an integrative metabonomic approach. *Chemical Research in Toxicology*, *14*, 1401–1412.

Wevers, R.A., Engelke, U., Wendel, U., de Jong, J.G.N., Gabreëls, F.J.M., Heerschap, A. (1995). Standardized method for high-resolution ^1^H-NMR of cerebrospinal fluid. *Clinical Chemistry*, *41(5),* 744–751.

Wishart, D.S. (2008). Quantitative metabolomics using NMR. *Trends in Analytical Chemistry*, *27(3),* 228–237.

Xia, J.; Psychogios, N., Young, N., Wishart, D.S. (2009). MetaboAnalyst: a web server for metabolomic data analysis and interpretation. *Nucleic Acids Research, 37* (Web Server issue): W652–60.

Xing, G., Ren, M., O’Neill, T.O., Sharma, P., Verma, A. (2012). Pyruvate dehydrogenase phosphatase 1 mRNA expression is divergently and dynamically regulated between rat cerebral cortex, hippocampus and thalamus after traumatic brain injury: A potential biomarker of TBI-induced hyper- and hypo-glycemia and neuronal vulnerability. *Neuroscience letters*, *525*, 140–145.
